# Projected distributions of 18 mosquito species in the Republic of Korea under climate change scenarios

**DOI:** 10.1186/s13071-026-07528-5

**Published:** 2026-06-21

**Authors:** Jihun Ryu, Kwang Shik Choi

**Affiliations:** 1 Vectorium Inc., Daegu, 111, Oksan-ro, Buk-gu, 41593 Republic of Korea; 2 Division of Foreign Animal Disease, Animal and Plant Quarantine Agency, Gimcheon, 39660 Republic of Korea; 3 BK21 FOUR KNU Creative BioResearch Group, School of Life Sciences, Kyungpook National University, Daegu, 41566 Republic of Korea

**Keywords:** Species distribution modeling, Climate change, Mosquito vectors, MaxEnt, Public health, Habitat suitability

## Abstract

**Background:**

Climate change is altering mosquito distributions and may reshape the risk of mosquito-borne diseases in the Republic of Korea (ROK). However, nationwide, multi-species projections for medically important mosquitoes remain limited.

**Methods:**

We compiled 1,969 spatially filtered occurrence records for 18 mosquito species and developed species distribution models using MaxEnt. Environmental predictors included bioclimatic, topographic, and land cover variables. Future distributions were projected for the 2030s, 2050s, and 2070s under SSP1-2.6, SSP2-4.5, and SSP5-8.5 scenarios using MIROC6 climate data. A Climate Change Vulnerability Index (CCVI) was calculated to compare species-specific range expansion and contraction.

**Results:**

Current habitat suitability patterns grouped the 18 species into nationwide, northern-preferring, southwestern-preferring, and southern-preferring distribution types. Topographic Wetness Index (36.6%), elevation (18.6%), and land cover type (18.4%) contributed more strongly to model performance than climatic variables alone. Under SSP5-8.5 in the 2070s, *Aedes albopictus* and *Culex tritaeniorhynchus* were projected to expand suitable habitat by 183.4% and 236.5%, respectively, whereas northern-preferring *Anopheles* species showed marked habitat contraction. *Anopheles pullus* and *Anopheles belenrae* were projected to lose 100.0% of their suitable habitat under the highest-emission scenario. The CCVI classified five species, mainly *Anopheles*, as highly vulnerable, one species as moderately affected, and 12 species as range-expanding.

**Conclusions:**

These findings indicate that climate change may substantially reorganize mosquito communities in the ROK, reducing habitat suitability for several malaria vectors while expanding suitable areas for important arbovirus and Japanese encephalitis vectors. Adaptive surveillance and vector management should therefore integrate climate projections with local landscape and hydrological risk factors.

**Supplementary Information:**

The online version contains supplementary material available at https://doi.org/10.1186/s13071-026-07528-5.

## Background

Mosquitoes rank among the most consequential disease vectors worldwide, annually exposing an estimated 700 million individuals to mosquito-transmitted infections and causing approximately 700,000 deaths [1]. Beyond direct mortality, mosquito-borne diseases impose substantial economic burdens, making them among the costliest infectious disease categories globally [2]. These insects transmit diverse pathogens responsible for malaria, dengue fever, Japanese encephalitis, yellow fever, West Nile fever, and Zika virus infection, threatening human health across all inhabited continents. Climate change and increasing global connectivity are fundamentally altering environmental conditions that govern mosquito distribution patterns and disease transmission dynamics.

Climate change affects mosquito ecology through multiple interacting mechanisms. Rising temperatures, shifting precipitation regimes, and altered humidity patterns influence mosquito developmental rates, reproductive output, survival, and capacity for pathogen transmission [3]. Temperature modulates mosquito development rate, biting frequency, and the extrinsic incubation period required for pathogen maturation, while precipitation determines the availability of breeding habitats [4, 5]. These climate-mediated effects operate through intricate feedback systems that may amplify or attenuate mosquito population responses depending on local environmental context. Research has documented geographic range expansion of key disease vectors, including *Aedes aegypti* and *Aedes albopictus*, into previously unsuitable territories [6, 7], whereas other species face habitat loss as temperatures surpass their physiological optima [8]. Characterizing these species-specific responses is fundamental for forecasting future disease risk and formulating effective management approaches.

Mosquito-borne diseases constitute significant public health challenges in the Republic of Korea (ROK), with a long epidemiological history. The ROK was historically endemic for malaria, with the disease reaching epidemic levels during the Korean War (1950–1953) when Plasmodium vivax transmission by Anopheles mosquitoes occurred throughout the peninsula [9, 10]. The National Malaria Eradication Service, established through WHO collaboration in 1959, implemented comprehensive control measures, including DDT application and antimalarial drug distribution, and successfully eliminated indigenous transmission by 1984. However, despite declaring malaria eradication in the 1970 s, the disease resurged in the late 1990 s near the Demilitarized Zone when a Korean soldier was diagnosed in 1993 [11]. Malaria transmission persists with annual incidence ranging from several hundred to over 2,000 cases, concentrated in regions where intensive vector suppression remains difficult due to geopolitical circumstances [12].

Japanese encephalitis represents another important component of the ROK's mosquito-borne disease history. Upon enactment of the Communicable Disease Control Act in 1954, Japanese encephalitis was designated a first-class notifiable disease, reflecting its status as a recurring public health concern with major outbreaks occurring at 2–3 year intervals throughout the 1950 s and 1960 s [13]. The most extensive recorded epidemic in 1958 involved 6897 reported cases. The introduction of the vaccination program in 1967 and its expansion nationally in 1983 fundamentally transformed the epidemiological situation. Although Japanese encephalitis incidence has declined substantially through vaccination, sporadic cases continue annually, with disease incidence increasing since 2010, predominantly affecting unvaccinated adults over 40 years [14]. The Korean Peninsula is experiencing warming rates that exceed the global average, potentially increasing susceptibility to changes in mosquito-borne disease risks [15]. Growing concern exists regarding potential establishment of emerging pathogens such as Zika, dengue, and chikungunya viruses, given climate change and enhanced global connectivity [16].

Approximately 60 mosquito species occur in the ROK, with over 10 classified as public health concerns [16]. Significant species include the *Anopheles hyrcanus* group, which serves as primary malaria vectors, comprising *Anopheles sinensis*, *Anopheles belenrae*, *Anopheles kleini*, *Anopheles lesteri*, and *Anopheles pullus* [17, 18]. Additional important species include *Culex tritaeniorhynchus*, the principal Japanese encephalitis vector that uses rice paddies as its primary breeding habitat [19]; the *Culex pipiens* complex, a West Nile virus vector common in urban environments [20]; and *Ae. albopictus*, a highly invasive urban-interface species with demonstrated competence for numerous arboviruses including dengue, chikungunya, and Zika [21]. These species have colonized diverse ecosystems across the ROK, exhibiting distinct ecological characteristics and habitat associations [22, 23].

Species Distribution Modeling (SDM) provides a quantitative framework for analyzing relationships between geographic distributions and environmental determinants to forecast potential habitats [24]. Grounded in ecological niche theory, these models characterize species' environmental requirements and tolerance limits, projecting them onto geographic maps to identify potential distribution areas. Early distribution models employed simple statistical methods, but advances in computing and Geographic Information Systems have enabled increasingly sophisticated algorithms [25]. Maximum Entropy (MaxEnt) modeling has gained widespread application for mosquito distribution prediction due to its effectiveness with presence-only data and capacity to capture complex nonlinear environment–occurrence relationships [26, 27]. MaxEnt estimates habitat suitability by minimizing relative entropy between probability densities in environmental space, effectively modeling intricate nonlinear associations and variable interactions [28]. This approach has been successfully applied to major vectors including *Ae. aegypti*, *Ae. albopictus*, and various *Anopheles* species across multiple geographic settings [29–33].

Mosquito ecological research in the ROK has predominantly focused on seasonal occurrence patterns, population density fluctuations, and species assemblages within specific localities through field surveillance using traps placed in disease-endemic or urban areas [23, 34, 35]. However, such field-based investigations face limitations in characterizing nationwide distribution patterns due to spatiotemporal constraints, irregular sampling-point distributions, and methodological heterogeneity across studies. Although individual investigations have examined specific mosquito species, comprehensive distribution modeling that encompasses all medically important mosquito species remains limited [36–38]. Moreover, comparative assessments of environmental factors that determine habitat suitability across species, and projections of distributional changes under multiple climate scenarios, remain insufficient. This knowledge gap constrains the development of evidence-based mosquito management strategies.

This study addresses these limitations through: (1) developing distribution models for 18 medically significant mosquito species in the ROK using 1,969 spatially filtered occurrence records; (2) systematically evaluating environmental factors affecting their distributions, including climatic, topographic, and land cover variables; (3) projecting future distributions under three Shared Socioeconomic Pathways scenarios (SSP1-2.6, SSP2-4.5, SSP5-8.5) for the 2030 s, 2050 s, and 2070 s; and (4) developing a Climate Change Vulnerability Index (CCVI) for quantitative evaluation of species-specific climate impacts. These findings provide essential scientific evidence to inform targeted mosquito surveillance and management strategies that are responsive to climate change in the ROK.

## Methods

### Study area and occurrence data

This investigation covered the entire ROK territory (approximately 100,449 km^2^), situated between 33°N and 38°N latitude and 124°E and 131°E longitude within the temperate climatic zone. This latitudinal extent encompasses substantial climatic gradients influencing mosquito species composition and seasonal activity. Mountains comprise roughly 70% of the total land area, with coastlines, plains, rivers, and wetlands creating diverse ecosystems providing varied mosquito habitats. The ROK experiences a characteristic temperate monsoon climate with distinct seasonal variation, featuring hot, humid summers and cold, dry winters.

Mosquito occurrence records were assembled from multiple sources: government surveillance programs (Korea Disease Control and Prevention Agency, Animal and Plant Quarantine Agency), Global Biodiversity Information Facility (GBIF) database, and published scientific literature. To minimize spatial autocorrelation, data were filtered using 1 km × 1 km resolution grids, retaining single-occurrence records when multiple coordinates existed within the same grid cell. Records were screened for geographic accuracy, temporal relevance (post-2000), and taxonomic reliability. The final dataset comprised 1,969 unique occurrence records across 18 species representing major medically important or frequently encountered mosquitoes in the ROK.

### Environmental variables

Environmental predictors included: (1) 19 bioclimatic variables from WorldClim version 2.1 [39]; (2) elevation data from CGIAR Consortium for Spatial Information; (3) Topographic Wetness Index (TWI) from Korea Institute of Geoscience and Mineral Resources; and (4) land cover classification (22 categories) from the Ministry of Environment. All variables were resampled to 1 km × 1 km resolution. Initial modeling incorporated all environmental variables, followed by an evaluation of each variable’s contribution for each species. Variables were ranked by contribution; among variables with Pearson correlation coefficients exceeding 0.85, only the highest-contributing variable was retained to minimize multicollinearity (Additional file 1: Fig. S1). Variables with coefficients below 0.1 were excluded, resulting in 8–11 environmental variables selected per species (Additional file 1: Table S1).

### Species distribution modeling

MaxEnt algorithm was implemented using 'dismo' [40] and 'ENMeval' [41] packages in R with MaxEnt software version 3.4.4 [26, 42]. To optimize model complexity and performance, regularization multipliers (RM) were evaluated from 0.5 to 5.0 in 0.5 increments (10 values), combined with six feature class (FC) combinations: Linear (L), Linear-Quadratic (LQ), Hinge (H), Linear-Quadratic-Hinge (LQH), Linear-Quadratic-Hinge-Product (LQHP), and Linear-Quadratic-Hinge-Product-Threshold (LQHPT), resulting in 60 candidate models per species. The corrected Akaike Information Criterion (AICc) was used for optimal model selection, with the model having a delta AICc value of 0 chosen as the final model [43]. Background points were generated using the default settings of ENMeval, which randomly samples approximately 10,000 points from the study area extent. No explicit bias correction was applied to background sampling, as spatial filtering of occurrence records (1 km × 1 km grid-based deduplication) was used to minimize sampling bias [44]. The entire study area was used as the accessible area for background sampling. Model performance was assessed using both the area under the receiver operating characteristic curve (AUC) and the True Skill Statistic (TSS). TSS is calculated as sensitivity + specificity − 1, ranging from − 1 to + 1, where values above 0.4 indicate good model performance and values above 0.8 indicate excellent performance [45]. For species with ≥ 50 occurrences, tenfold cross-validation was conducted; leave-one-out validation was applied for species with < 50 records. Continuous suitability maps were converted to binary presence/absence maps using a fixed threshold of 0.5, where pixels with predicted suitability ≥ 0.5 were classified as suitable habitat. This threshold was used consistently across all species for calculating suitable habitat area, range change metrics, and the Climate Change Vulnerability Index.

### Future distribution projection

Future distributional shifts were projected using three Shared Socioeconomic Pathways scenarios: SSP1-2.6 (sustainable development, reduced emissions), SSP2-4.5 (intermediate pathway), and SSP5-8.5 (fossil fuel-intensive, high emissions) [46]. Future climate data utilized MIROC6 global climate model [47] bioclimatic variables for 2021–2040 (2030s), 2041–2060 (2050s), and 2061–2080 (2070s) from WorldClim. Models calibrated under current conditions were applied for future projections, updating only bioclimatic variables. Non-climate variables (elevation, TWI, land cover) were held constant to isolate the direct effects of climate change on mosquito distributions.

### Climate change vulnerability index

A climate change vulnerability index (CCVI) was formulated to quantitatively assess species-specific climate vulnerability. Binary presence/absence maps (suitability threshold ≥ 0.5) were generated for both current and each future scenario. For each comparison, pixels were classified as: range expansion (pixels unsuitable in the current period but suitable in the future period), range contraction (pixels suitable in the current period but unsuitable in the future period), or range stable (pixels suitable in both periods). The expansion and contraction ratios were calculated as the proportion of pixels that expanded or contracted relative to the current suitable area. By normalizing to the current suitable area, these ratios account for differences in the extent of baseline distribution among species, enabling direct cross-species comparison. The CCVI was then computed as the average expansion ratio minus the average contraction ratio across all scenarios (SSP1-2.6, SSP2-4.5, SSP5-8.5) and time periods (2030s, 2050 s, 2070 s). This formulation captures the net balance between range gain and range loss in a single metric: positive values indicate net habitat gain (range expansion) and negative values indicate net habitat loss (high vulnerability). We adopted a difference-based metric rather than a ratio-based metric (e.g., expansion/contraction) because differences allow symmetric interpretation on a continuous scale, where zero represents no net change, and values can be directly compared across species regardless of whether the dominant process is expansion or contraction. Species were classified into three categories: range expansion (CCVI > 0.5), moderate impact (−0.5 ≤ CCVI ≤ 0.5), and high vulnerability (CCVI < −0.5).

## Results

### Model performance and environmental variable contributions

MaxEnt distribution models were successfully constructed for all 18 mosquito species using 1969 spatially filtered occurrence records and optimized environmental variable sets (Fig. 1). The modeling framework captured both broad-scale climatic controls and fine-scale habitat requirements that govern mosquito distributions across Korea's heterogeneous landscapes. All models demonstrated satisfactory to excellent discrimination, with mean AUC of 0.857 (± 0.029 SD), ranging from 0.799 (*Ae. albopictus*) to 0.907 (*Oc. togoi*), and a mean TSS of 0.578 (± 0.059 SD), ranging from 0.474 (*An. sinensis*) to 0.683 (*Cx. vagans*) (Table 1). All TSS values exceeded 0.4, confirming statistically significant predictive power across all species models [45]. Detailed MaxEnt distribution maps and model diagnostics for each species are provided in Additional file 1: Figures S3–S20, with environmental response curves shown in Additional file 1: Figures S21–S38.

**Fig. 1:**
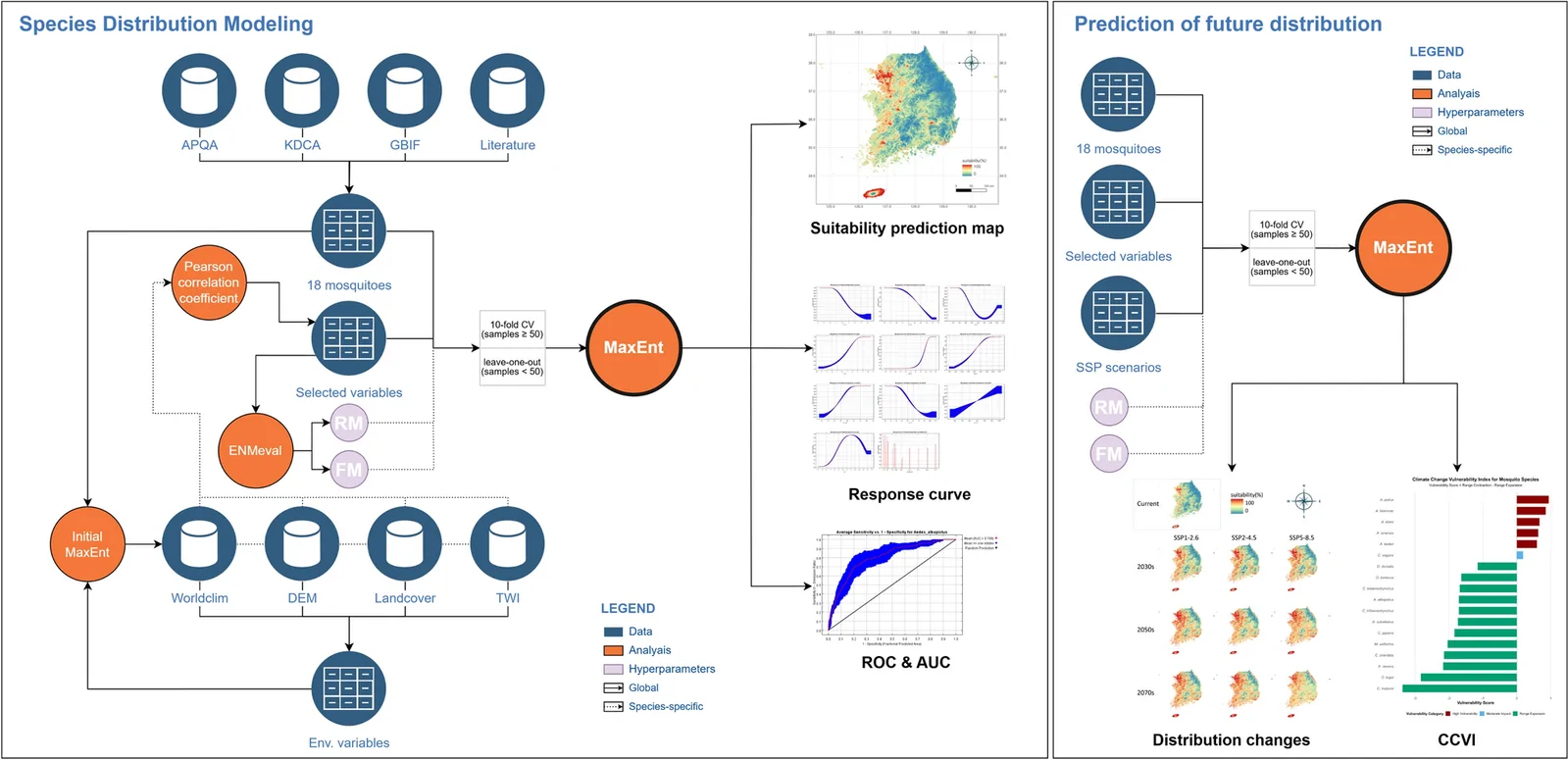
Methodological framework for species distribution modeling. The workflow illustrates data collection from multiple sources (APQA, KDCA, GBIF, Literature), environmental variable selection through Pearson correlation filtering and ENMeval hyperparameter optimization, MaxEnt model development with cross-validation, and future distribution prediction under three SSP scenarios (SSP1-2.6, SSP2-4.5, SSP5-8.5). Key outputs include suitability prediction maps, response curves, ROC/AUC validation, and Climate Change Vulnerability Index assessment

**Table 1:** MaxEnt model performance metrics and optimal hyperparameters for 18 mosquito species in the Republic of Korea

Species	No. occurrence records	AUC value	TSS	Optimal RM	Optimal FC	SD	Training AUC – test AUC
*Aedes albopictus*	216	0.799	0.517	2.0	LQ	0.047	0.026
*Aedes vexans*	159	0.869	0.564	1.5	LQ	0.038	0.015
*Anopheles belenrae*	57	0.845	0.565	3.0	LQ	0.108	0.025
*Anopheles kleini*	58	0.844	0.601	5.0	LQH	0.115	0.018
*Anopheles lesteri*	31	0.895	0.642	3.5	L	0.112	0.017
*Anopheles pullus*	70	0.889	0.653	2.5	L	0.057	0.013
*Anopheles sinensis*	197	0.801	0.474	3.0	LQH	0.035	0.020
*Armigeres subalbatus*	162	0.859	0.577	3.0	LQH	0.031	0.017
*Culex bitaeniorhynchus*	94	0.849	0.602	3.5	LQ	0.028	0.020
*Culex inatomii*	57	0.881	0.663	5.0	LQ	0.051	0.022
*Culex orientalis*	120	0.864	0.552	1.0	LQ	0.035	0.018
*Culex pipiens*	212	0.827	0.494	1.0	LQ	0.021	0.027
*Culex tritaeniorhynchus*	158	0.846	0.565	1.5	LQ	0.048	0.026
*Culex vagans*	48	0.892	0.683	4.5	LQH	0.130	0.016
*Mansonia uniformis*	70	0.873	0.653	1.5	LQ	0.060	0.026
*Ochlerotatus dorsalis*	137	0.850	0.543	1.0	LQ	0.042	0.022
*Ochlerotatus koreicus*	163	0.839	0.559	2.5	LQ	0.018	0.013
*Ochlerotatus togoi*	62	0.907	0.513	1.5	LQ	0.051	0.027

*AUC*, area under the curve; *TSS*, true skill statistic; *FC*, feature class; *RM*, regularization multiplier; *SD,* standard deviation. *TSS* is calculated as sensitivity + specificity − 1

These metrics compare favorably with previous mosquito modeling studies, indicating that environmental variables successfully captured major distributional determinants. The relatively lower AUC for *Ae. albopictus* likely reflects its widespread distribution and broad ecological tolerance. Cross-validation demonstrated model stability with a mean training–test AUC difference of only 0.020 (± 0.005), indicating minimal overfitting.

Environmental variable contribution analysis revealed 8–11 variables retained per species model following selection procedures designed to eliminate redundant predictors while maintaining ecological interpretability. An average of 9.6 variables were included per species model, comprising climatic (bioclimatic), topographic (elevation, TWI), and land cover variables. Primary factors determining mosquito distributions in the ROK were TWI (36.6% mean contribution), elevation (18.6%), land cover (18.4%), bio10 (mean temperature of warmest quarter, 15.9%), and bio15 (precipitation seasonality, 11.4%) (Table 2). Notably, topographic and landscape variables had a greater influence than climatic variables, suggesting that local-scale habitat characteristics play crucial roles in mosquito distribution patterns. The prominence of TWI likely reflects its mechanistic relationship to breeding-habitat water availability.

**Table 2:** Environmental variable contributions (%) to species distribution models

Env. variable type	Selection frequency	Avg. contribution (%)	Range (%)
bio1	1	1.6	
bio2	13	5.1	0.1–22.5
bio3	16	1.3	0.0–5.1
bio4	18	4.7	0.0–34.2
bio5	1	7.1	
bio6	13	2.3	0.3–10.1
bio7	0		
bio8	1	9.1	
bio9	2	7.7	0.0–15.3
bio10	9	15.9	6.2–26.3
bio11	1	0.8	
bio12	3	3.8	0.1–10.7
bio13	7	1.7	0.0–3.7
bio14	12	0.7	0.0–3.7
bio15	16	11.4	0.1–60.3
bio16	5	2.1	0.9–3.2
bio17	0		
bio18	6	2.8	0.0–7.0
bio19	6	9.9	0.0–41.2
DEM	7	18.6	12.2–27.6
LC	18	18.4	0.0–33.5
TWI	18	36.6	3.1–60.7

bio variables from WorldClim database; *DEM*, Digital Elevation Model; *LC*, land cover; *TWI*, Topographic Wetness Index

### Current distribution patterns and habitat characteristics

**Fig. 2:**
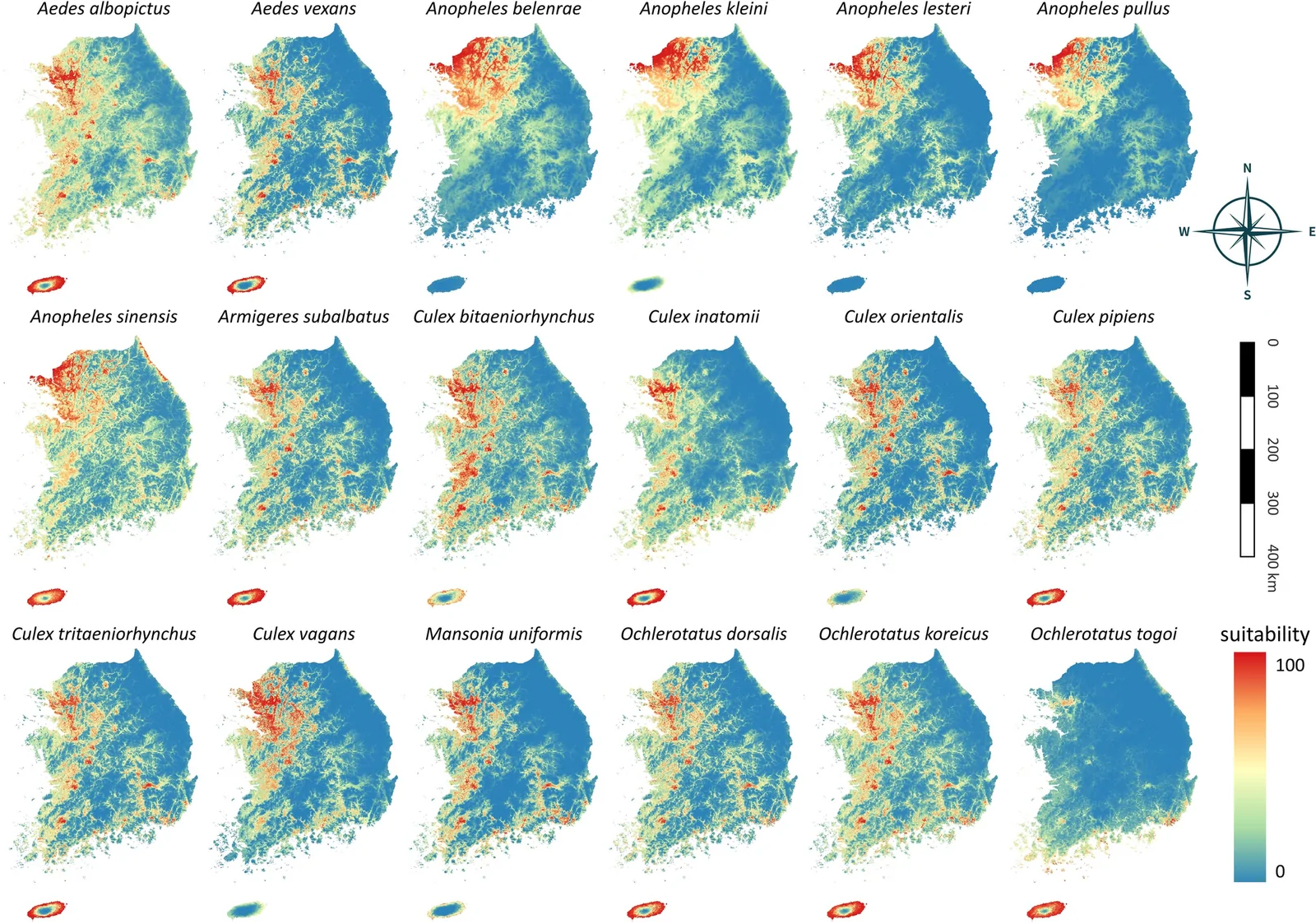
Current habitat suitability maps for 18 mosquito species in the Republic of Korea. Suitability values range from 0 (blue, unsuitable) to 100 (red, highly suitable). Species are arranged alphabetically. Scale bar indicates 400 km

Predicted habitat distributions for 18 mosquito species exhibited distinct patterns, with species grouped into four categories based on geographic range and suitability levels (Fig. 2). Species were classified through visual assessment of habitat suitability maps, combined with quantitative analyses of the geographical centroid and extent of suitable habitats (suitability ≥ 0.5). The nationwide distribution type included 11 species: *Ae. albopictus*, *Ae. vexans*, *An. sinensis*, *Ar. subalbatus*, *Cx. bitaeniorhynchus*, *Cx. orientalis*, *Cx. pipiens* complex, *Cx. tritaeniorhynchus*, *Cx. vagans*, *Oc. dorsalis*, and *Oc. koreicus*, showing habitat suitability across the peninsula except the northeastern mountainous regions. Notably, *Ae. albopictus* and *An. sinensis* showed suitable habitats covering 20.2% and 21.5% of the total national area, respectively, with average suitability of 31.2% and 30.7%.

Northern-preferring species included *An. belenrae*, *An. kleini*, *An. lesteri*, and *An. pullus*, exhibiting high suitability primarily in regions north of 37°N latitude. This pattern suggests adaptation to cooler temperatures and potential evolutionary origins in continental climates. These *Anopheles* Hyrcanus group members serve as primary malaria vectors in the ROK, and their northern preference corresponds to the geographic concentration of malaria cases near the DMZ. Southwestern-preferring species included *Mansonia uniformis* and *Culex inatomii*, showing affinity for warmer coastal regions. The southern-preferring type was uniquely represented by *Oc. togoi*, concentrated along coastlines and on Jeju Island.

Land cover analysis across all species' habitats revealed the highest suitability in artificial grassland (20.8%), residential areas (13.6%), dry fields (11.3%), deciduous forests (7.5%), transportation areas (7.3%), commercial areas (6.4%), and rice paddies (4.5%) (Additional file 1: Fig. S2). Mosquito species exhibited elevated suitability in urban and peri-urban environments with high human activity. This pattern highlights mosquito adaptation to anthropogenic habitats. Southwestern-preferring *Cx. inatomii* and *M. uniformis* preferred the lowest elevations (median 24 m and 22 m, respectively), while *Anopheles* species generally favored higher elevations (median 41–113 m). The only southern-preferring species, *Oc. togoi*, exhibited both wider and higher elevation range (4–413 m), likely due to the concentration of high-suitability habitats on topographically varied Jeju Island (Table S2).

### Projected distribution changes under climate scenarios

**Fig. 3:**
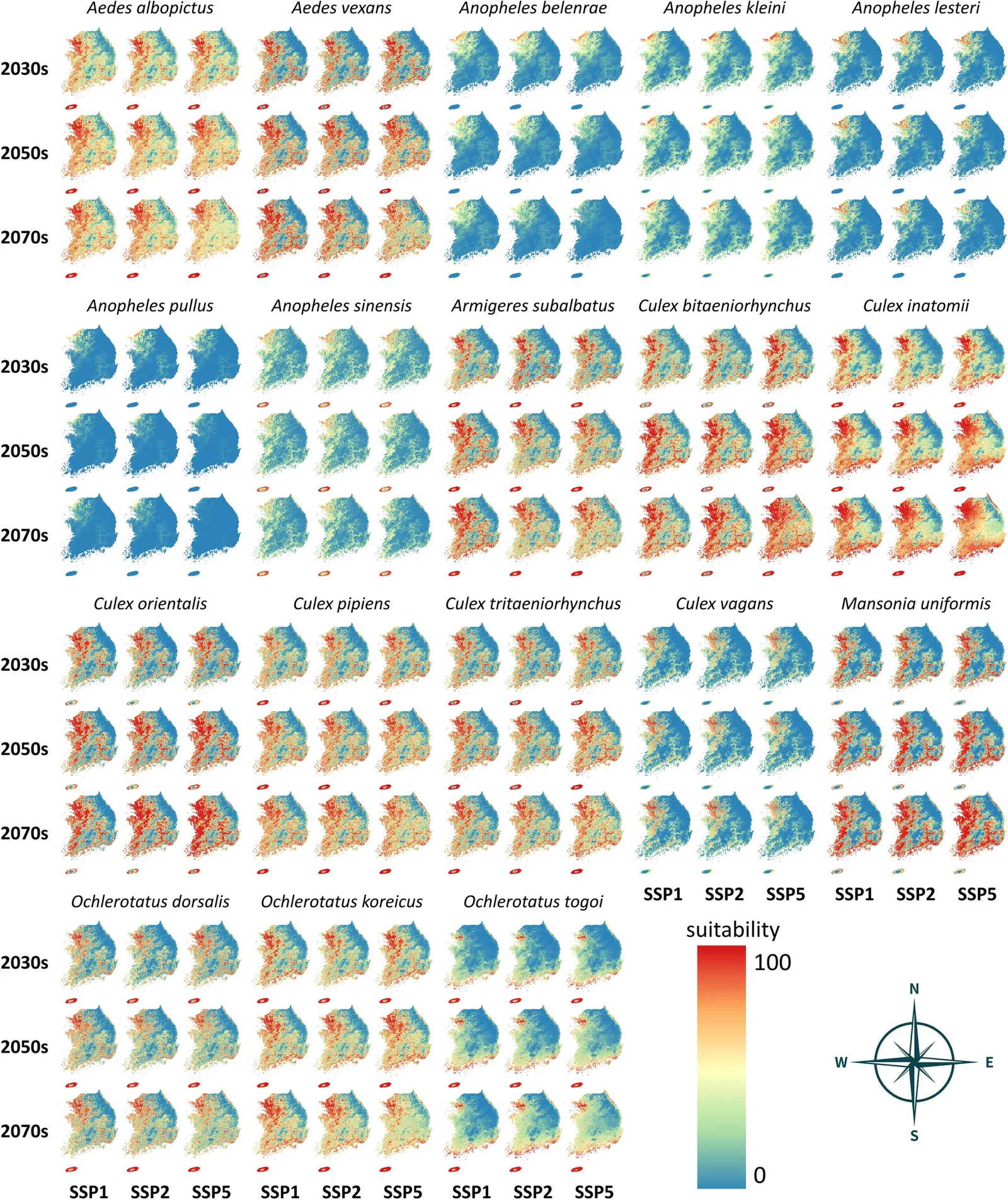
Predicted distribution changes of 18 mosquito species under different SSP scenarios (SSP1-2.6, SSP2-4.5, SSP5-8.5) for the 2030 s, 2050 s, and 2070s. Suitability values range from 0 (blue) to 100 (red). Each row represents a different time period, and each column represents a different SSP scenario

Future distribution analysis for 18 species across the 2030 s (2021–2040), 2050 s (2041–2060), and 2070 s (2061–2080) using MIROC6 SSP scenario data predicted substantial climate-driven impacts varying by scenario, time period, and species ecology (Fig. 3; Table 3). Change magnitude and direction reflect species-specific thermal tolerances and current positions relative to optimal conditions. The most dramatic changes were projected under SSP5-8.5, where a 4–5 °C temperature increase by century's end would generate fundamentally novel climatic conditions that would exceed historical variability for many species.

**Table 3:** Changes in habitat suitability (suitable area %) by SSP scenario and time period

Species		SSP1-2.6			SSP2-4.5			SSP5-8.5		
	Current	2030s	2050s	2070s	2030s	2050s	2070s	2030s	2050s	2070s
*Aedes albopictus*	31.21 (20.24)	46.65 (44.56)	55.93 (59.65)	54.65 (58.33)	46.95 (44.79)	52.07 (54.29)	56.56 (61.06)	48.95 (47.88)	57.85 (65.05)	55.46 (57.37)
*Aedes vexans*	20.33 (14.07)	39.35 (37.67)	47.73 (47.40)	50.40 (50.26)	36.50 (34.07)	44.86 (44.24)	48.97 (47.98)	41.95 (39.95)	51.19 (52.38)	50.22 (47.96)
*Anopheles belenrae*	22.30 (15.41)	11.48 (3.79)	9.51 (2.35)	10.13 (2.26)	11.80 (4.03)	8.89 (1.88)	7.52 (0.74)	11.71 (4.41)	6.94 (0.57)	3.61 (0)
*Anopheles kleini*	23.77 (12.03)	18.20 (5.37)	16.99 (4.18)	16.70 (4.00)	17.92 (5.17)	16.76 (3.78)	16.06 (3.22)	17.36 (5.34)	15.74 (2.76)	15.26 (2.21)
*Anopheles lesteri*	16.51 (10.07)	11.02 (4.78)	10.52 (4.68)	10.88 (4.67)	11.16 (4.92)	9.69 (3.90)	10.22 (4.10)	10.81 (4.78)	9.07 (3.21)	7.48 (2.06)
*Anopheles pullus*	13.83 (8.74)	5.01 (1.13)	2.70 (0.25)	3.61 (0.44)	5.82 (1.45)	2.83 (0.22)	3.35 (0.22)	4.15 (0.84)	1.87 (0.02)	0.60 (0)
*Anopheles sinensis*	30.74 (21.48)	20.32 (8.53)	19.39 (7.78)	18.55 (6.66)	21.16 (10.25)	21.12 (9.95)	19.24 (8.24)	20.74 (9.82)	17.59 (5.86)	14.79 (4.19)
*Armigeres subalbatus*	22.74 (15.00)	37.61 (34.21)	48.46 (47.70)	50.29 (49.58)	37.16 (33.36)	41.62 (38.49)	44.21 (40.62)	39.33 (36.53)	44.43 (41.84)	50.46 (47.29)
*Culex bitaeniorhynchus*	25.92 (18.47)	40.00 (35.52)	53.43 (52.34)	50.10 (48.82)	37.01 (31.70)	50.44 (49.21)	57.27 (57.45)	44.89 (42.18)	57.23 (57.84)	66.47 (70.89)
*Culex inatomii*	20.95 (12.18)	43.83 (41.94)	53.22 (55.38)	54.83 (57.04)	39.46 (35.56)	50.19 (50.90)	57.41 (60.75)	44.67 (42.94)	58.01 (62.25)	64.56 (71.77)
*Culex orientalis*	19.60 (13.21)	37.64 (34.79)	43.06 (40.86)	44.36 (41.93)	33.12 (29.67)	42.53 (40.44)	50.92 (49.27)	36.10 (33.55)	48.74 (46.75)	57.65 (56.49)
*Culex pipiens*	24.30 (16.03)	38.48 (36.14)	47.44 (46.62)	49.27 (48.89)	38.48 (36.01)	47.10 (46.47)	52.40 (53.05)	41.98 (39.98)	50.11 (49.90)	53.78 (52.60)
*Culex tritaeniorhynchus*	21.66 (14.97)	37.90 (35.03)	44.50 (42.43)	43.60 (41.38)	35.11 (31.84)	40.13 (37.43)	45.17 (42.74)	37.07 (34.06)	50.25 (49.55)	52.39 (50.37)
*Culex vagans*	21.03 (15.16)	18.47 (12.37)	20.51 (14.99)	20.91 (15.35)	17.10 (10.85)	17.78 (11.50)	19.95 (14.04)	17.87 (11.68)	17.12 (10.79)	16.21 (9.48)
*Mansonia uniformis*	18.38 (12.96)	33.06 (31.30)	37.89 (36.74)	41.96 (40.78)	31.68 (29.72)	42.23 (41.22)	45.66 (44.75)	33.21 (31.56)	44.44 (43.88)	54.61 (54.54)
*Ochlerotatus dorsalis*	22.29 (14.99)	33.98 (30.57)	39.00 (36.24)	38.95 (35.41)	29.45 (24.45)	32.84 (27.96)	40.39 (37.41)	36.27 (33.13)	35.32 (30.07)	40.96 (35.59)
*Ochlerotatus koreicus*	25.41 (16.91)	42.88 (39.59)	47.43 (45.83)	51.03 (51.30)	39.13 (34.26)	44.89 (42.56)	49.93 (49.19)	44.65 (42.36)	52.31 (53.24)	48.47 (43.29)
*Ochlerotatus togoi*	10.73 (4.83)	20.74 (13.07)	27.13 (19.22)	25.39 (17.17)	22.36 (14.65)	26.31 (18.12)	32.81 (24.50)	23.29 (15.05)	28.34 (19.30)	35.66 (25.59)

Values represent percentage of total national area with predicted suitability values ≥ 0.5. Suitable habitat area was determined by applying a fixed binary threshold of 0.5 to continuous suitability predictions

Among nationwide species, nine showed substantial habitat expansion, with some extending ranges across much of the peninsula. *Culex bitaeniorhynchus* increased from 18.5 to 70.9% of the suitable area by the 2070 s under SSP5-8.5, representing a 284% increase. *Cx. orientalis* expanded from 13.2 to 56.5%, a 328% increase. Both species are potential vectors of Japanese encephalitis, suggesting that climate change may expand the geographic risk area for this disease. Conversely, *An. sinensis*, the most widespread malaria vector regionally, declined from 21.5 to 4.2% suitable area, an 80% reduction. Key disease vectors *Ae. albopictus* and *Cx. tritaeniorhynchus* showed expansion from 20.2 to 57.4% (183.4% increase) and from 15.0 to 50.4% (236.5% increase), respectively. *Ae. albopictus* expansion is particularly concerning given its demonstrated vector competence for multiple arboviruses (Table 4).

**Table 4:** Classification of future distribution change patterns by species under climate change

Species		Suitable area change (%)	Suitability change (%)
Expanding species	*Aedes albopictus*	+ 183.4	+ 77.7
	*Aedes vexans*	+ 240.9	+ 147.1
	*Armigeres subalbatus*	+ 215.3	+ 121.9
	*Culex bitaeniorhynchus*	+ 283.8	+ 156.5
	*Culex inatomii*	+ 489.2	+ 208.2
	*Culex orientalis*	+ 327.6	+ 194.1
	*Culex pipiens*	+ 228.1	+ 121.3
	*Culex tritaeniorhynchus*	+ 236.5	+ 141.8
	*Mansonia uniformis*	+ 320.8	+ 197.2
	*Ochlerotatus dorsalis*	+ 137.4	+ 83.7
	*Ochlerotatus koreicus*	+ 156.0	+ 90.8
	*Ochlerotatus togoi*	+ 429.8	+ 232.2
Contracting species	*Anopheles belenrae*	− 100.0	− 83.8
	*Anopheles kleini*	− 99.3	− 54.7
	*Anopheles lesteri*	− 96.6	− 35.8
	*Anopheles pullus*	− 100.0	− 68.7
	*Anopheles sinensis*	− 80.5	− 33.1
Stable species	*Culex vagans*	− 37.5	− 22.9

Change calculated for 2070 s under SSP5-8.5 scenario relative to current conditions

Northern-preferring species showed rapidly declining suitable habitats, with several facing potential extirpation from the peninsula. *Anopheles pullus* declined from 8.7 to 0% suitable area under SSP5-8.5 for the 2070 s, suggesting complete habitat loss from the ROK. *An. belenrae* similarly declined from 15.4 to 0%. *An. kleini* decreased from 10.5 to 0.1%, and *An. lesteri* from 7.6 to 0.3%. These dramatic declines in *Anopheles* spp. populations have significant implications for vivax malaria epidemiology. Even under lower-emission SSP1-2.6, substantial reductions were projected for these species.

Southwestern-preferring species showed substantially expanded suitable habitats, benefiting from warming conditions. *M. uniformis* increased from 13.0 to 54.5% (321% increase), and *Cx. inatomii* from 12.2 to 71.8% (489.2% increase). Southern-preferring *Oc. togoi* increased from 4.8 to 25.6% (429.8% increase), indicating a dramatic expansion from a primarily coastal distribution to inland areas. Temporal analysis revealed that most expanding species showed continuous increases in suitability throughout the 2030 s, 2050 s, and 2070 s, with the most dramatic increases under SSP5-8.5.

### Climate change vulnerability index assessment

**Fig. 4:**
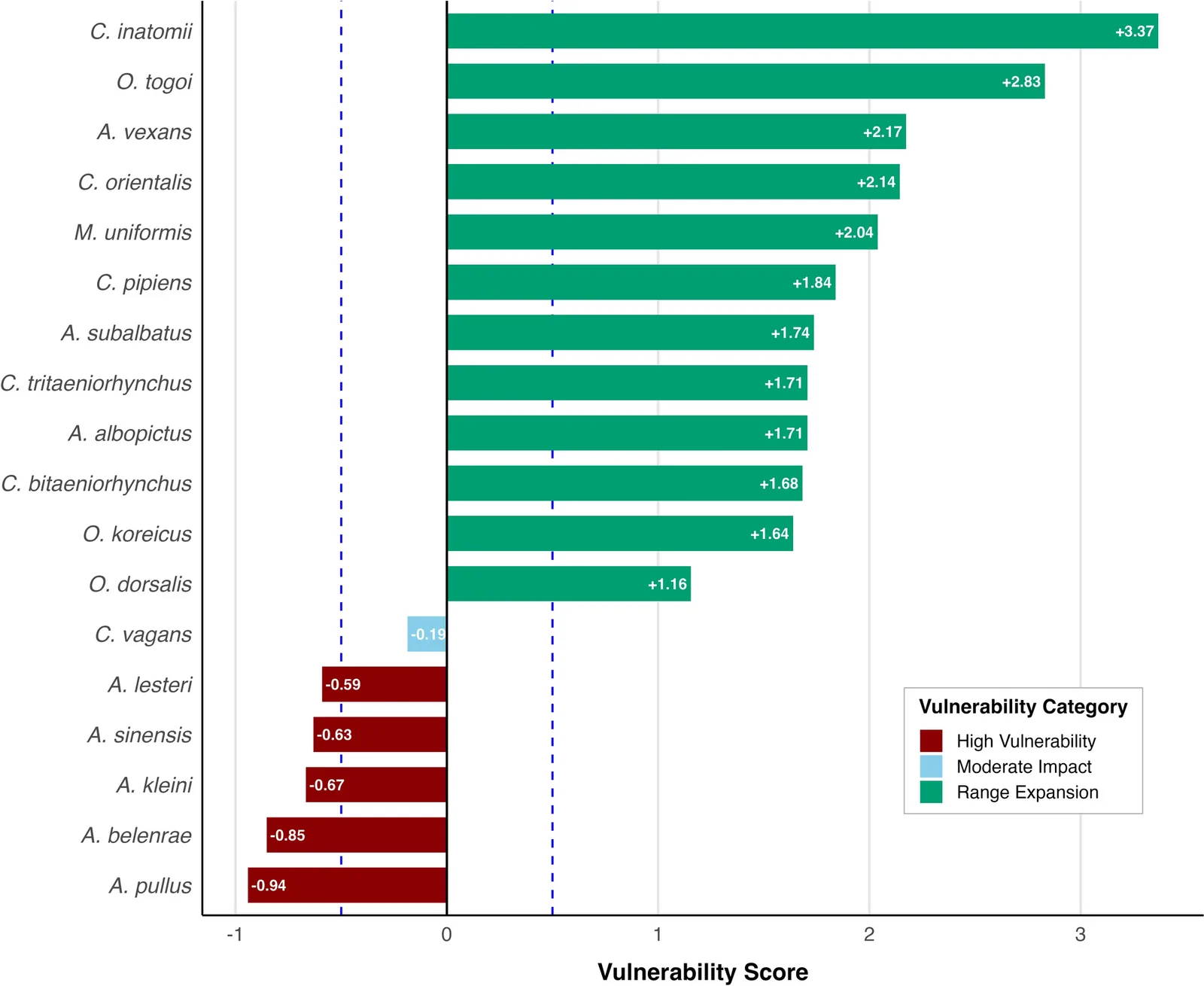
Climate Change Vulnerability Index (CCVI) for 18 mosquito species in the Republic of Korea, calculated as the average range expansion ratio minus the average range contraction ratio across all climate scenarios (SSP1-2.6, SSP2-4.5, SSP5-8.5) and time periods (2030s, 2050 s, 2070 s). Positive values (green bars) indicate net range expansion under future climate conditions, while negative values (red bars) indicate net range contraction (high vulnerability). Light blue indicates moderate impact. Dashed lines indicate classification thresholds (± 0.5). Species are ordered by CCVI value from most vulnerable (bottom) to greatest expansion (top)

CCVI analysis classified 18 species into three groups: high vulnerability (five species, primarily *Anopheles*), moderate impact (one species, *Cx. vagans* with CCVI = − 0.19), and range expansion (12 species) (Fig. 4). The most vulnerable species were *An. pullus* (CCVI = − 0.94) and *An. belenrae* (CCVI = − 0.85), indicating near-complete projected habitat loss. *An. kleini* (CCVI = − 0.67), *An. sinensis* (CCVI = − 0.63), and *An. lesteri* (CCVI = − 0.59) also showed high vulnerability. All highly vulnerable species belong to the *Anopheles hyrcanus* group, suggesting climate change may substantially reduce malaria vector populations in the ROK.

Species showing the greatest expansion included *Cx. inatomii* (CCVI =  + 3.37) and *Oc. togoi* (CCVI =  + 2.83), both of which are currently restricted to southern and coastal areas. Other substantially expanding species included *Ae. vexans* (CCVI =  + 2.17), *Cx. orientalis* (CCVI =  + 2.14), *M. uniformis* (CCVI =  + 2.04), *Cx. pipiens* (CCVI =  + 1.84), *Ar. subalbatus* (CCVI =  + 1.74), and *Cx. tritaeniorhynchus* (CCVI =  + 1.71). These range-expanding species include multiple disease vectors, indicating climate change may alter vector community composition and associated disease risks.

## Discussion

This study presents the first comprehensive multi-species distribution modeling analysis for 18 medically important mosquito species in the ROK, yielding novel insights into environmental determinants of mosquito distributions and projected climate change impacts. Our findings demonstrate that topographic and landscape factors, rather than climatic variables, were the primary determinants of distribution. TWI emerged as the most influential predictor (36.6%), followed by elevation (18.6%) and land cover (18.4%). This finding contrasts with numerous global studies that emphasize climate as the primary driver of mosquito distributions [6, 7], suggesting that landscape-level habitat management may be particularly effective in the Korean context. The limited climatic influence may reflect South Korea's climate already satisfying breeding requirements for most species throughout much of the year, with greater spatial differentiation arising from landscape characteristics. While our future projections altered only bioclimatic variables because landscape changes are inherently difficult to predict, the substantial contribution of non-climatic factors to current distributions suggests that landscape-level management, such as controlling water accumulation and modifying land-use practices, may serve as an important complementary strategy for mitigating climate-driven changes in vector distributions.

The prominence of TWI aligns with growing recognition of hydrology's significance in mosquito ecology [48]. TWI integrates topographic position and catchment area to predict soil moisture patterns, providing a mechanistically grounded measure of water availability superior to precipitation alone. These variables capture both permanent breeding sites and temporary water accumulations crucial for mosquito reproduction. The mechanistic basis of TWI provides confidence that models incorporating this variable will remain applicable under changing conditions, as topographic controls on water accumulation are relatively stable over climate-projection timescales.

Land cover strongly influenced habitat suitability, with artificial grasslands (20.8%) and residential areas (13.6%) showing the highest suitability across species. This aligns with research indicating that mosquitoes prefer environments that balance vegetation resources with proximity to human dwellings [21, 49]. These interface habitats provide optimal conditions that combine water sources, shelter, and blood meal hosts, while often lacking the natural predators found in undisturbed areas. Elevated suitability in artificial grasslands may result from regular irrigation, which creates temporary water pools and reduces predator pressure compared to natural wetlands. Urban areas provide diverse microhabitats, including artificial containers, underground infrastructure, and irrigated green spaces, supporting mosquito breeding [50]. This pattern emphasizes mosquito adaptation to anthropogenic environments and the critical role of human-modified landscapes in determining vector populations.

The four distinct distribution patterns (nationwide, northern-preferring, southwestern-preferring, and southern-preferring) reflect species-specific thermal tolerances and ecological requirements. Northern-preferring Anopheles species appear to operate near their thermal limits under current conditions, thereby explaining their high climate-change vulnerability. Previous investigations have documented the importance of temperature thresholds for *Anopheles* spp. development and malaria transmission [51, 52]. Potential complete habitat loss for *An. pullus* and *An. belenrae* under high-emission scenarios has significant implications for vivax malaria epidemiology, as these species are primary vectors in the DMZ region [17, 18]. However, the relationship between vector population decline and disease transmission is complex and may not yield proportional reductions in malaria incidence if remaining populations increase their relative contribution to transmission.

Conversely, a substantial projected expansion of *Ae. albopictus* (183.4% increase) and *Cx. tritaeniorhynchus* (236.5% increase) raises significant public health concerns. *Aedes albopictus* is highly invasive and has demonstrated the capacity to transmit numerous arboviruses, including dengue, chikungunya, and Zika [53], while *Cx. tritaeniorhynchus* serves as the primary Japanese encephalitis vector in East Asia [19]. Global projections have similarly predicted range expansion for these vectors under climate change [54, 55]. These findings suggest that while malaria risk may decrease, the risk of Japanese encephalitis and arboviral diseases transmitted by *Aedes* species may increase substantially in the ROK. This shifting disease risk profile necessitates adaptive surveillance strategies accounting for emerging threats from expanding species.

The CCVI developed here provides a standardized metric for comparing climate impacts across species and informing management priorities. Classification into high vulnerability, moderate impact, and range expansion categories offers a framework for prioritizing surveillance and control efforts. Species in the expansion category (positive CCVI values), particularly disease vectors like *Ae. albopictus* and *Cx. tritaeniorhynchus* warrant enhanced monitoring in newly suitable areas [56]. That all five highly vulnerable species (negative CCVI values) belong to the Anopheles Hyrcanus group suggests a coherent response pattern linked to shared ecological requirements, rendering this group particularly sensitive to warming.

Several methodological considerations merit discussion. First, our models assumed non-climate variables (elevation, TWI, land cover) remain constant under future projections. While this assumption permits the isolation of climate effects, land-use changes accompanying urbanization and agricultural intensification may substantially modify habitat availability independently of climate [57]. It is important to note that, because topographic variables were held constant, species whose distributions are heavily influenced by topographic factors may show more conservative range shifts than would occur if landscape changes were also incorporated. Second, while MaxEnt is robust for presence-only data, it cannot account for biotic interactions, dispersal limitations, or adaptive responses to environmental change [58, 59]. The models assume unlimited dispersal capacity, meaning that any area predicted as climatically suitable is considered potentially occupiable regardless of dispersal barriers. In reality, mosquito dispersal is constrained by flight range (typically 0.1–3 km for most species), geographic barriers, and habitat connectivity. For the ROK, the geographic constraint of being bordered by North Korea to the north means that northward range shifts by expanding species may extend beyond the study area, whereas contracting species cannot retreat farther north. Additionally, evolutionary adaptation and phenotypic plasticity could allow species to persist in areas predicted to become unsuitable, and novel competitive interactions may emerge as species distributions shift. Third, our analysis employed a single global climate model (MIROC6) rather than an ensemble of multiple GCMs. MIROC6 was selected because it is one of the primary models used in IPCC assessment reports and has demonstrated strong performance in simulating East Asian climate patterns [47]. While using a single GCM allows for clearer interpretation of scenario-specific projections, it does not capture the uncertainty arising from structural differences among climate models. Future studies should incorporate multi-model ensembles to quantify inter-model uncertainty. Fourth, no explicit bias correction was applied to background point generation beyond spatial filtering of occurrence records at 1 km resolution. While this approach reduces spatial autocorrelation, it may not fully account for geographic biases in mosquito surveillance efforts. Despite these limitations, the consistency of our findings with known species’ ecological characteristics and their alignment with broader patterns in global mosquito studies support the validity of our approach.

These findings carry important implications for mosquito-borne disease management in the ROK. Identification of TWI and land cover as primary distributional determinants suggests local-scale interventions targeting water accumulation areas and habitat modification may prove effective in the near term. Projected shifts in vector species composition indicate the need for adaptive surveillance strategies that account for emerging risks posed by expanding species while maintaining vigilance for currently important vectors. Future research should incorporate additional climate models, consider land-use change scenarios, and validate predictions against field surveillance data to refine these projections.

## Conclusions

This study provides the first nationwide multi-species assessment of current and future habitat suitability for 18 medically important mosquito species in the ROK under climate change scenarios. The results show that topographic and landscape factors, particularly TWI, elevation, and land cover, are major determinants of current mosquito distributions, while future warming is projected to drive divergent species-specific responses. Northern-preferring *Anopheles* species, including *An. pullus*, *An. belenrae*, *An. kleini*, *An. sinensis*, and *An. lesteri*, are expected to undergo substantial habitat contraction, whereas several medically important range-expanding species, including *Ae. albopictus*, *Cx. tritaeniorhynchus*, *Cx. inatomii*, and *Oc. togoi*, may gain suitable habitat under future climate conditions. These projected shifts suggest that the disease-risk profile in the ROK may change from one dominated by historically important malaria vectors toward increasing concern for Japanese encephalitis and arboviral disease vectors. Vector surveillance and control programs should therefore be adapted to account for both climate-driven range shifts and local habitat determinants, with particular attention to newly suitable areas for expanding species and continued monitoring of vulnerable vector populations.

## Supplementary Information


Additional file 1.

## Data Availability

Environmental variables are publicly accessible from WorldClim (https://www.worldclim.org/), CGIAR-CSI (https://srtm.csi.cgiar.org/), and Korea Institute of Geoscience and Mineral Resources.
