# Supplementary material for: Projected distributions of 18 mosquito species in the Republic of Korea under climate change scenarios
Source: Parasit Vectors. 2026 Jun 21;19:375. doi: 10.1186/s13071-026-07528-5 (PMC13531818; doi:10.1186/s13071-026-07528-5)
Supplement: Supplementary file 1 [file 13071_2026_7528_MOESM1_ESM.pdf]

Supplementary Materials

Projected distributions of 18 mosquito species in the Republic of Korea under climate change scenarios

Supplementary Tables

Table S1. Final environmental variables selected for each mosquito species distribution model.

Species	No. Env. variables	Major Env. variables
Aedes albopictus	10	bio2, bio3, bio4, bio6, bio10, bio13, bio15, bio19, landcvover, twi
Aedes dorsalis	10	bio2, bio3, bio4, bio6, bio10, bio13, bio14, bio15, landcvover, twi
Aedes koreicus	11	bio2, bio3, bio4, bio6, bio10, bio12, bio14, bio15, bio16, landcvover, twi
Aedes togoi	9	bio2, bio4, bio6, bio10, bio12, bio14, bio16, landcvover, twi
Aedes vexans	10	bio2, bio3, bio4, bio6, bio10, bio14, bio15, bio18, landcover, twi
Anopheles belenrae	9	bio3, bio4, bio6, bio15, bio18, bio19, dem, landcover, twi
Anopheles kleini	9	bio2, bio4, bio11, bio13, bio15, bio19, dem, landcover, twi
Anopheles lesteri	8	bio3, bio4, bio15, bio18, bio19, dem, landcover, twi
Anopheles pullus	9	bio3, bio4, bio6, bio13, bio15, bio19, dem, landcover, twi
Anopheles sinensis	10	bio2, bio3, bio4, bio6, bio13, bio15, bio19, dem, landcover, twi
Armigeres subalbatus	10	bio2, bio3, bio4, bio6, bio10, bio14, bio15, bio16, landcover, twi
Culex bitaeniorhynchus	10	bio2, bio3, bio4, bio5, bio6, bio14, bio15, bio16, landcover, twi
Culex inatomii	9	bio2, bio3, bio4, bio6, bio10, bio14, bio18, landcover, twi
Culex orientalis	10	bio2, bio3, bio4, bio6, bio8, bio13, bio14, bio15, landcover, twi
Culex pipiens	10	bio2, bio3, bio4, bio6, bio10, bio13, bio14, bio15, landcover, twi
Culex tritaeniorhynchus	11	bio2, bio3, bio4, bio9, bio10, bio12, bio14, bio15, bio18, landcover, twi
Culex vagans	9	bio3, bio4, bio9, bio14, bio15, bio18, dem, landcover, twi
Mansonia uniformis	9	bio1, bio3, bio4, bio14, bio15, bio16, dem, landcover, twi

Table S2. Elevation suitability summary for major mosquito species (suitability threshold ≥0.75).

Species	Optimal elevation range	Optimal elevation range (IQR) (m)	Elevation (median) (m)
Aedes albopictus	6 - 245	20 - 68	37
Aedes dorsalis	7 - 216	25 - 72	43
Aedes koreicus	6 - 225	21 - 66	38
Aedes togoi	4 - 413	26 - 148	63
Aedes vexans	7 - 146	22 - 60	37
Anopheles belenrae	7 - 255	28 - 132	67
Anopheles kleini	9 - 283	48 - 183	113
Anopheles lesteri	6 - 142	20 - 72	41
Anopheles pullus	6 - 217	25 - 111	59
Anopheles sinensis	7 - 258	20 - 99	45
Armigeres subalbatus	8 - 286	23 - 73	41
Culex bitaeniorhynchus	4 - 104	14 - 44	25
Culex inatomii	4 - 229	13 - 49	24
Culex orientalis	9 - 132	26 - 68	43
Culex pipiens	7 - 244	25 - 76	43
Culex tritaeniorhynchus	6 - 162	20 - 60	35
Culex vagans	7 - 120	22 - 69	41
Mansonia uniformis	3 - 70	12 - 36	22
Mean	6 - 208	23 - 83	45

Supplementary Figures

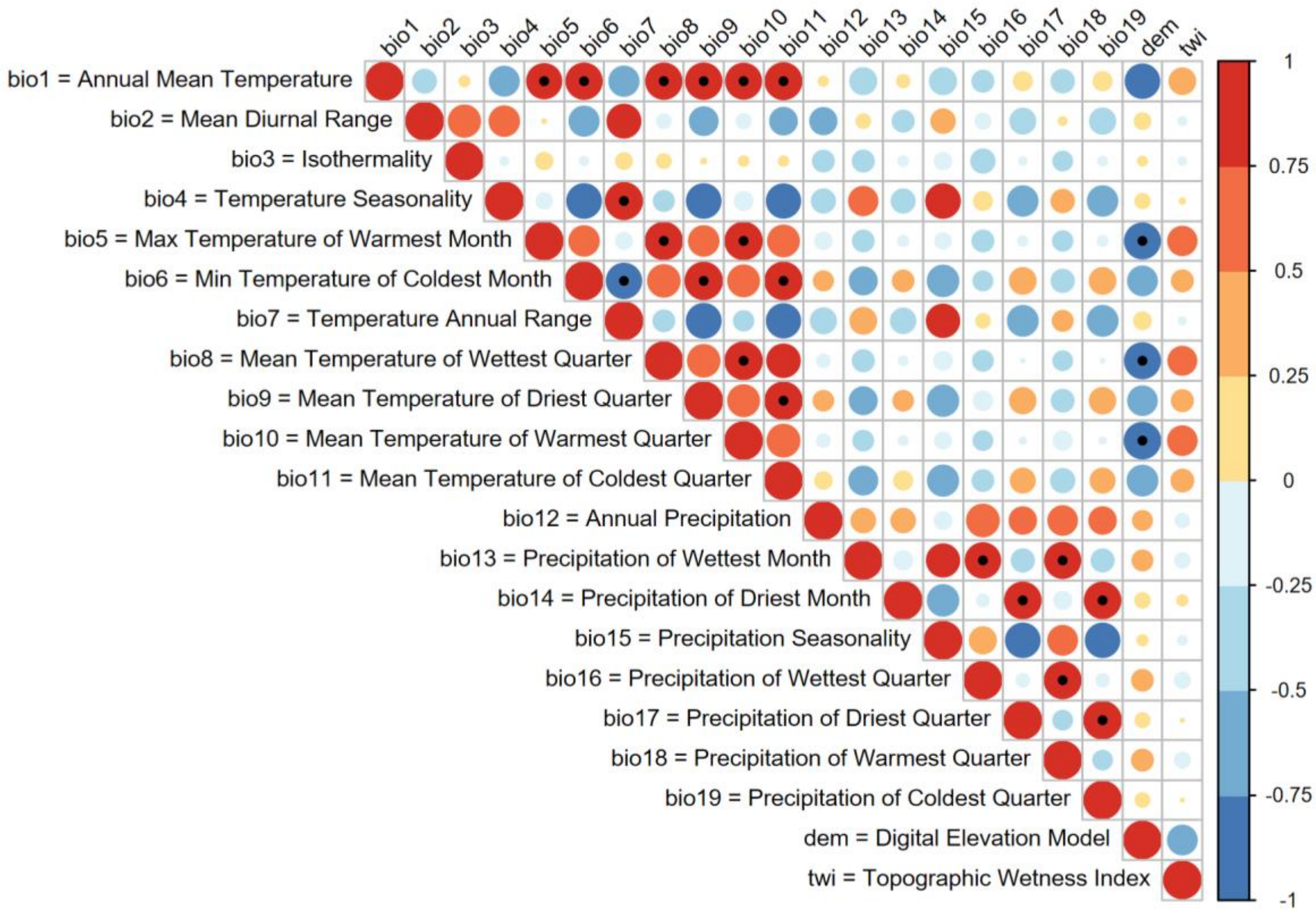

Figure S1. Correlation matrix of environmental variables (black dots indicate correlation coefficients ≥0.85).

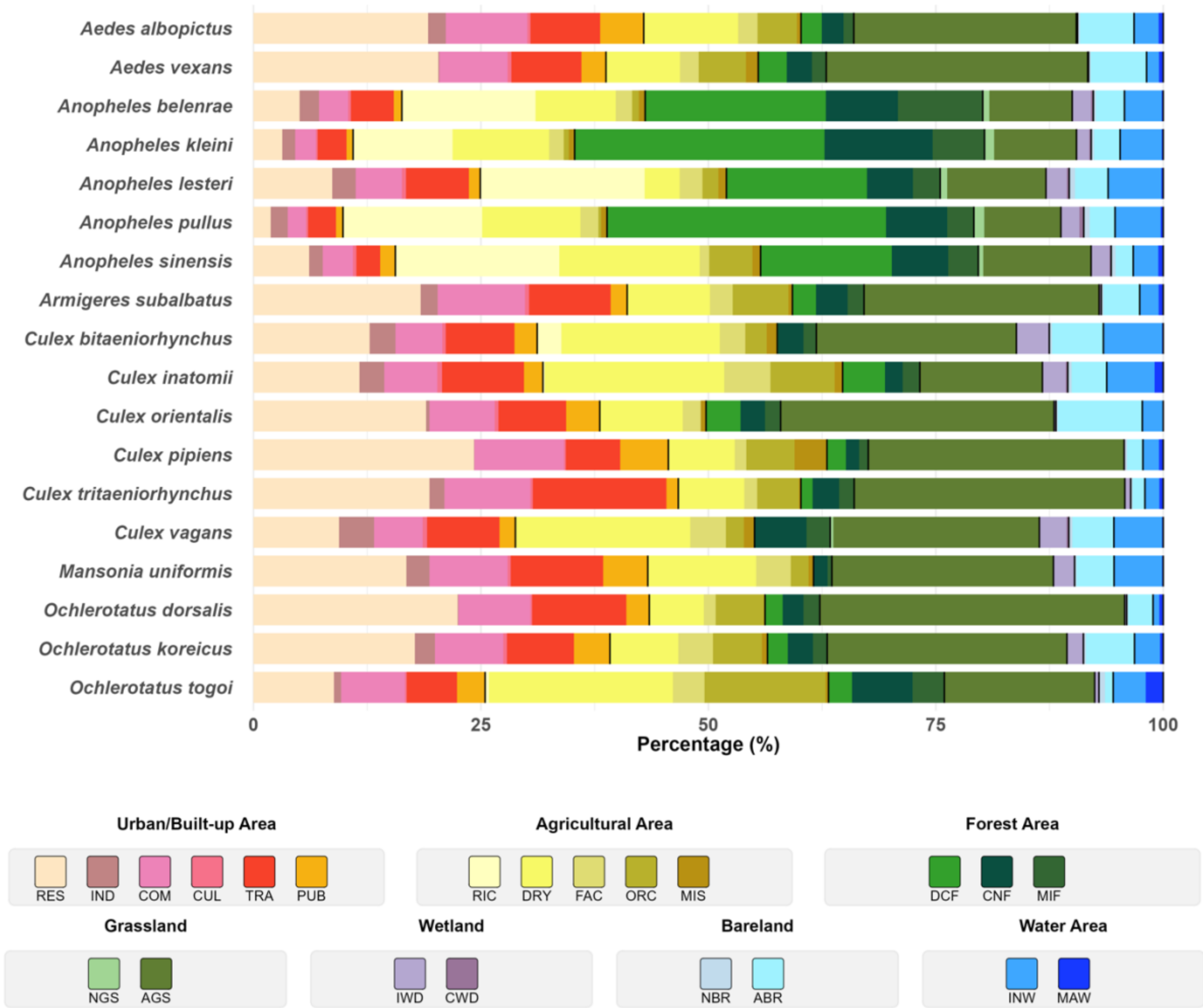

Figure S2. Preferred land cover types extracted from the land cover environmental variable using MaxEnt results (suitability threshold ≥0.75).

Individual Species MaxEnt Distribution Results

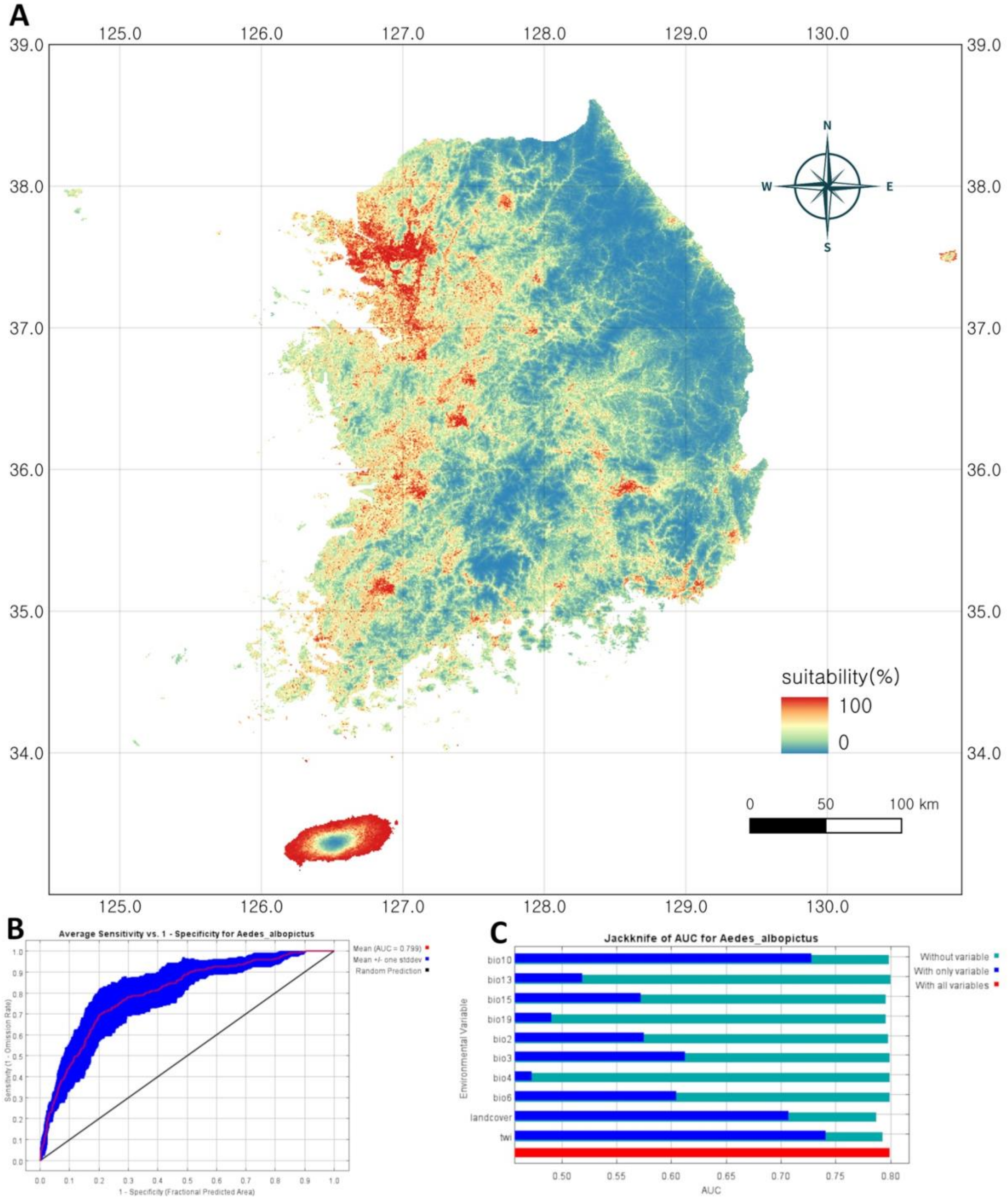

Figure S3. MaxEnt species distribution model results for Aedes albopictus. (A) Current predicted habitat suitability map for the Republic of Korea. Warmer colors (red) indicate higher predicted suitability and cooler colors (blue) indicate lower suitability (range: 0-100%). (B) Receiver operating characteristic (ROC) curve showing model discrimination ability (mean AUC = 0.799). (C) Jackknife test of variable importance based on AUC. The final model used 10 environmental variables (Table S1) with optimal regularization multiplier = 2.0 and linear-quadratic (LQ) feature class.

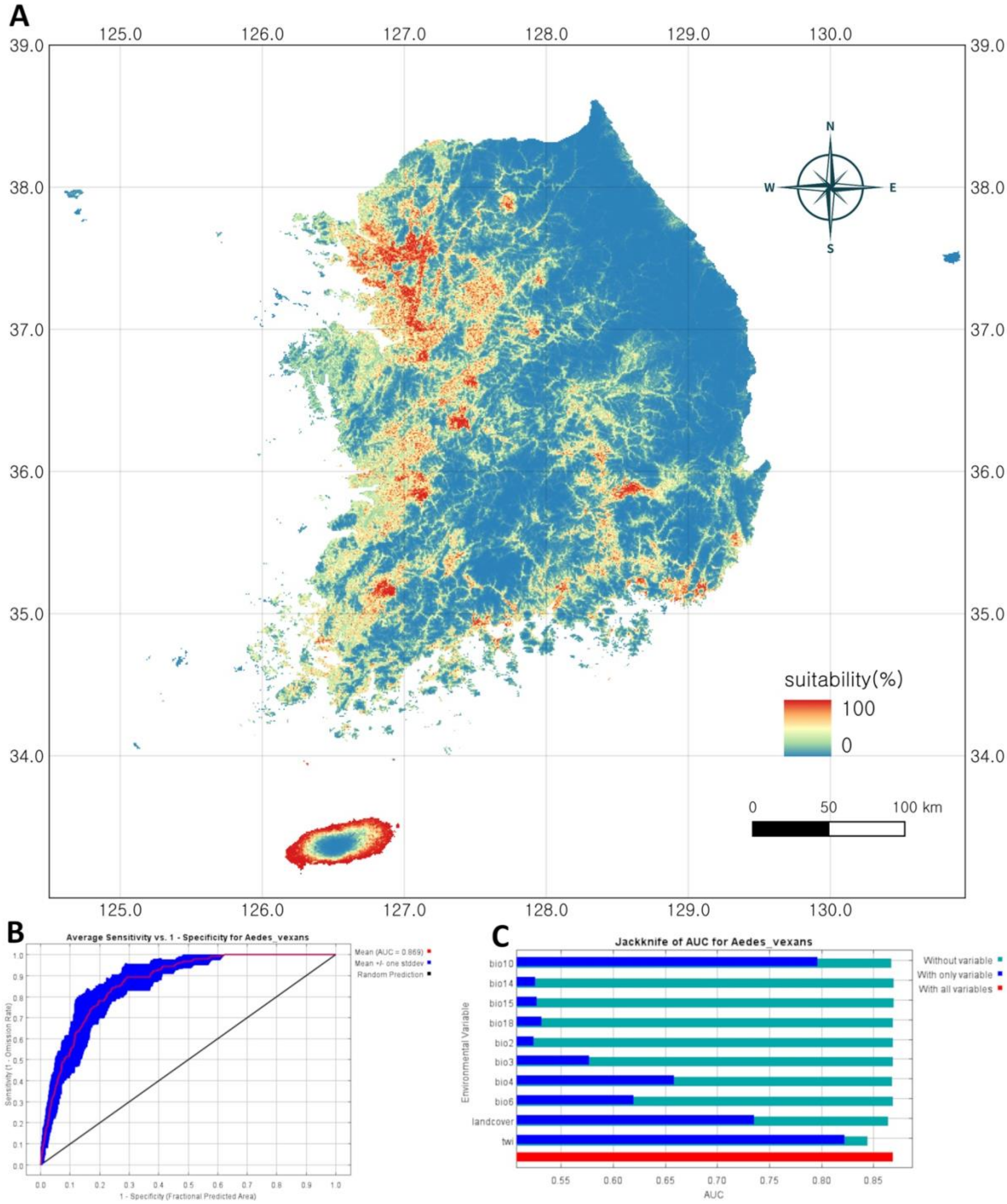

Figure S4. MaxEnt species distribution model results for Aedes vexans. (A) Current predicted habitat suitability map for the Republic of Korea. Warmer colors (red) indicate higher predicted suitability and cooler colors (blue) indicate lower suitability (range: 0-100%). (B) Receiver operating characteristic (ROC) curve showing model discrimination ability (mean AUC = 0.869). (C) Jackknife test of variable importance based on AUC. The final model used 10 environmental variables (Table S1) with optimal regularization multiplier = 1.5 and linear-quadratic (LQ) feature class.

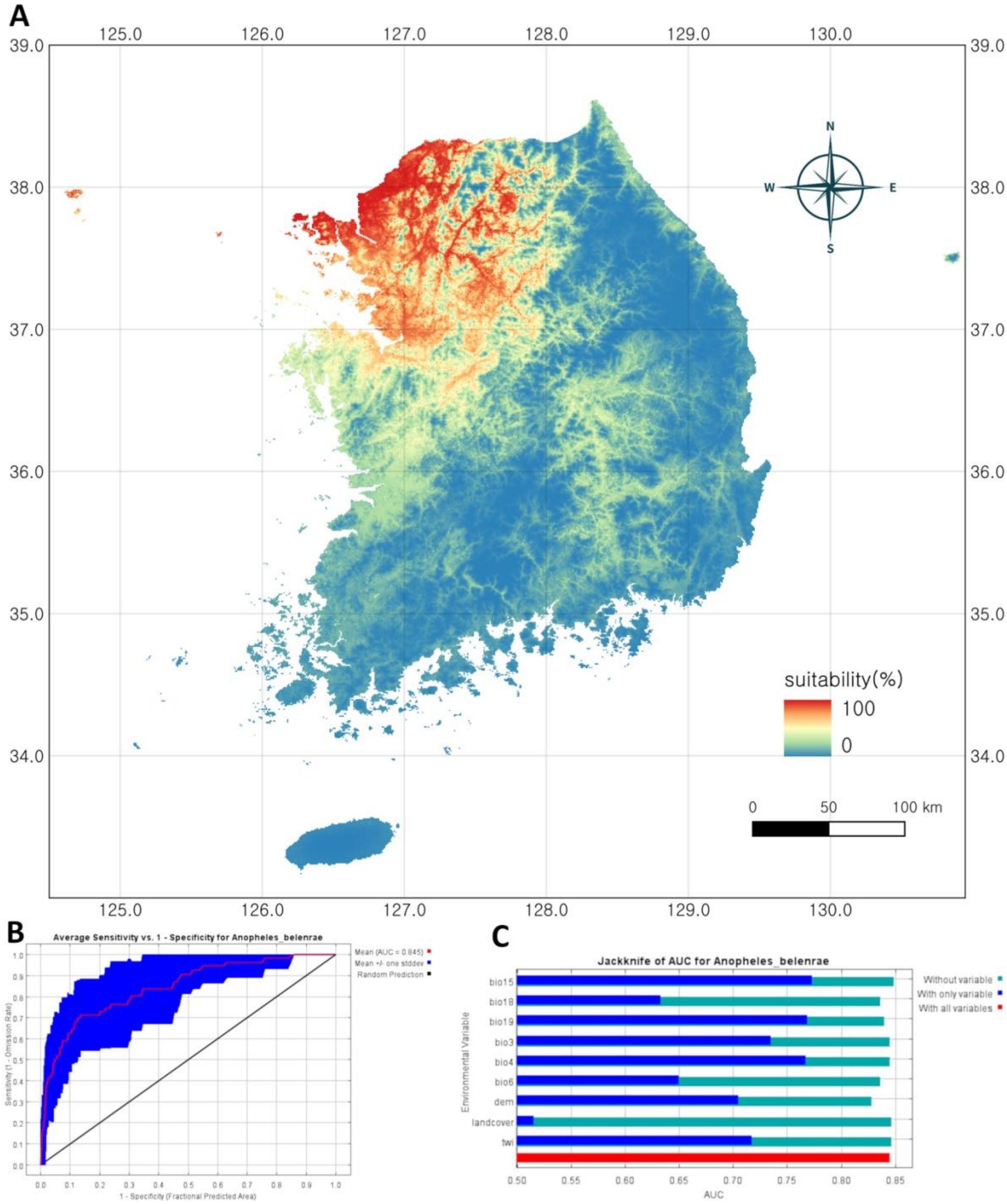

Figure S5. MaxEnt species distribution model results for Anopheles belenrae. (A) Current predicted habitat suitability map for the Republic of Korea showing a northern-preferring distribution pattern. Warmer colors (red) indicate higher predicted suitability and cooler colors (blue) indicate lower suitability (range: 0-100%). (B) Receiver operating characteristic (ROC) curve showing model discrimination ability (mean AUC = 0.845). (C) Jackknife test of variable importance based on AUC. The final model used 9 environmental variables (Table S1) with optimal regularization multiplier = 3.0 and linear-quadratic (LQ) feature class.

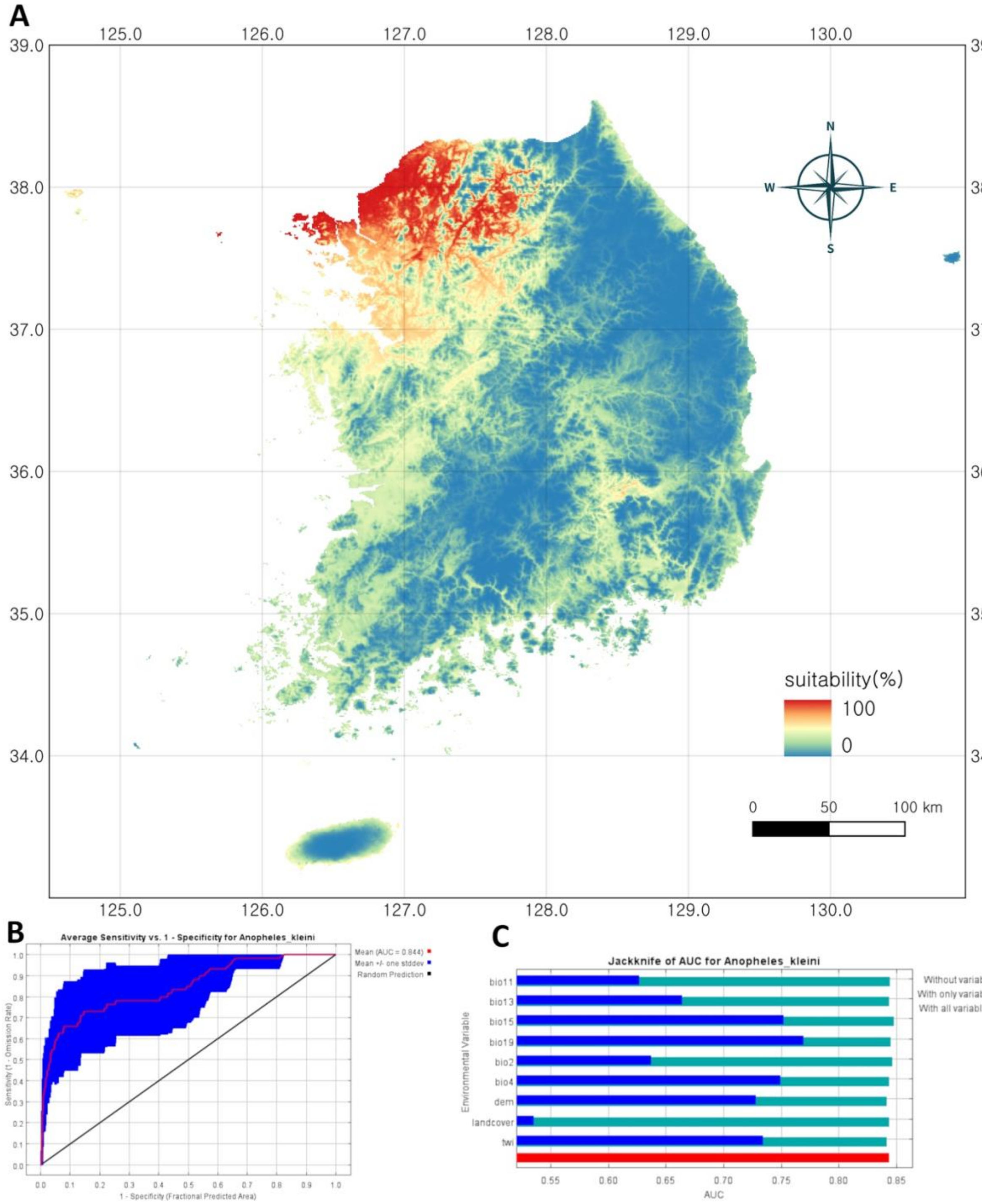

Figure S6. MaxEnt species distribution model results for Anopheles kleini. (A) Current predicted habitat suitability map for the Republic of Korea showing a northern-preferring distribution pattern. Warmer colors (red) indicate higher predicted suitability and cooler colors (blue) indicate lower suitability (range: 0-100%). (B) Receiver operating characteristic (ROC) curve showing model discrimination ability (mean AUC = 0.844). (C) Jackknife test of variable importance based on AUC. The final model used 9 environmental variables (Table S1) with optimal regularization multiplier = 5.0 and linear-quadratic-hinge (LQH) feature class.

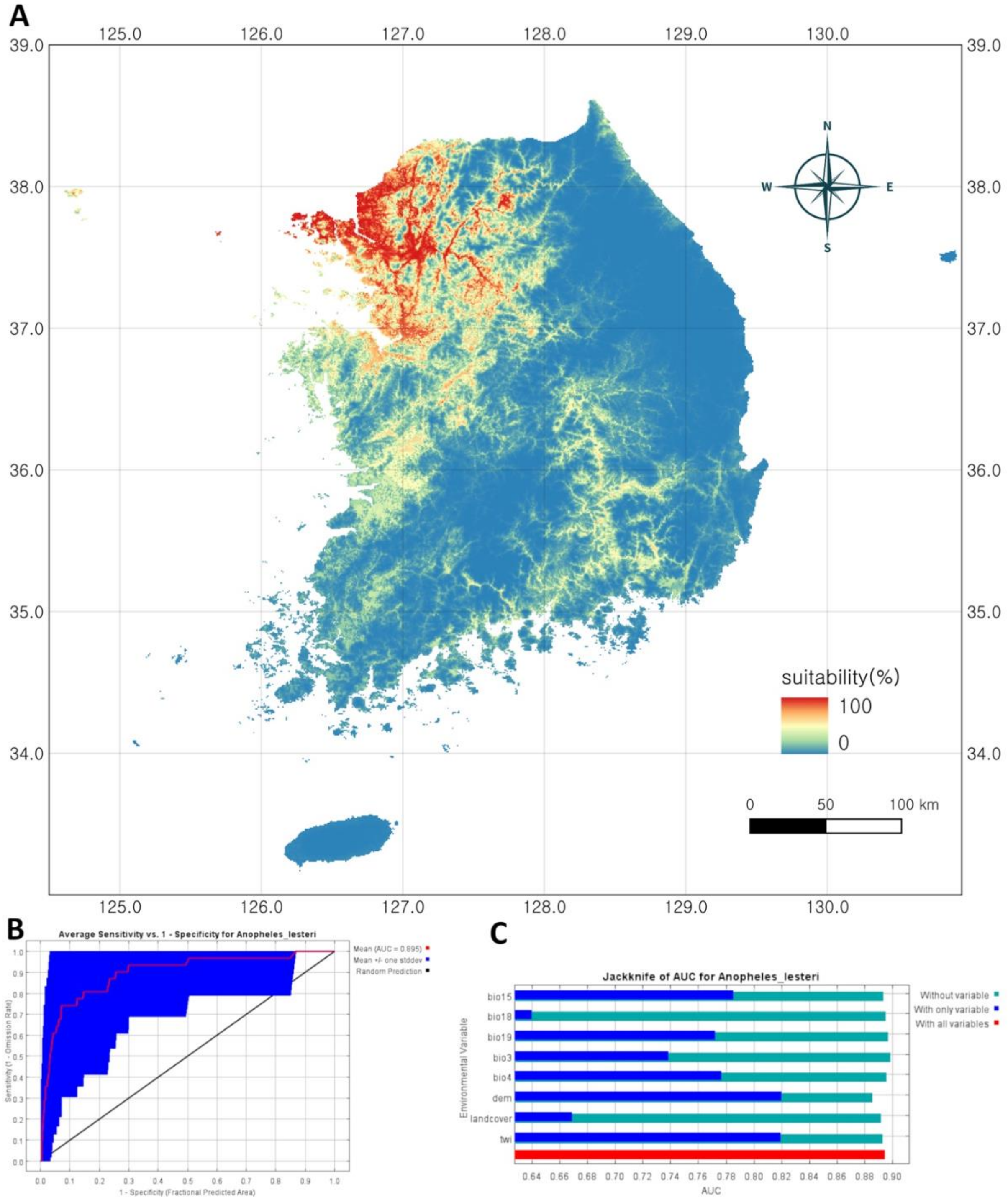

Figure S7. MaxEnt species distribution model results for Anopheles lesteri. (A) Current predicted habitat suitability map for the Republic of Korea showing a northern-preferring distribution pattern. Warmer colors (red) indicate higher predicted suitability and cooler colors (blue) indicate lower suitability (range: 0-100%). (B) Receiver operating characteristic (ROC) curve showing model discrimination ability (mean AUC = 0.895). (C) Jackknife test of variable importance based on AUC. The final model used 8 environmental variables (Table S1) with optimal regularization multiplier = 3.5 and linear (L) feature class.

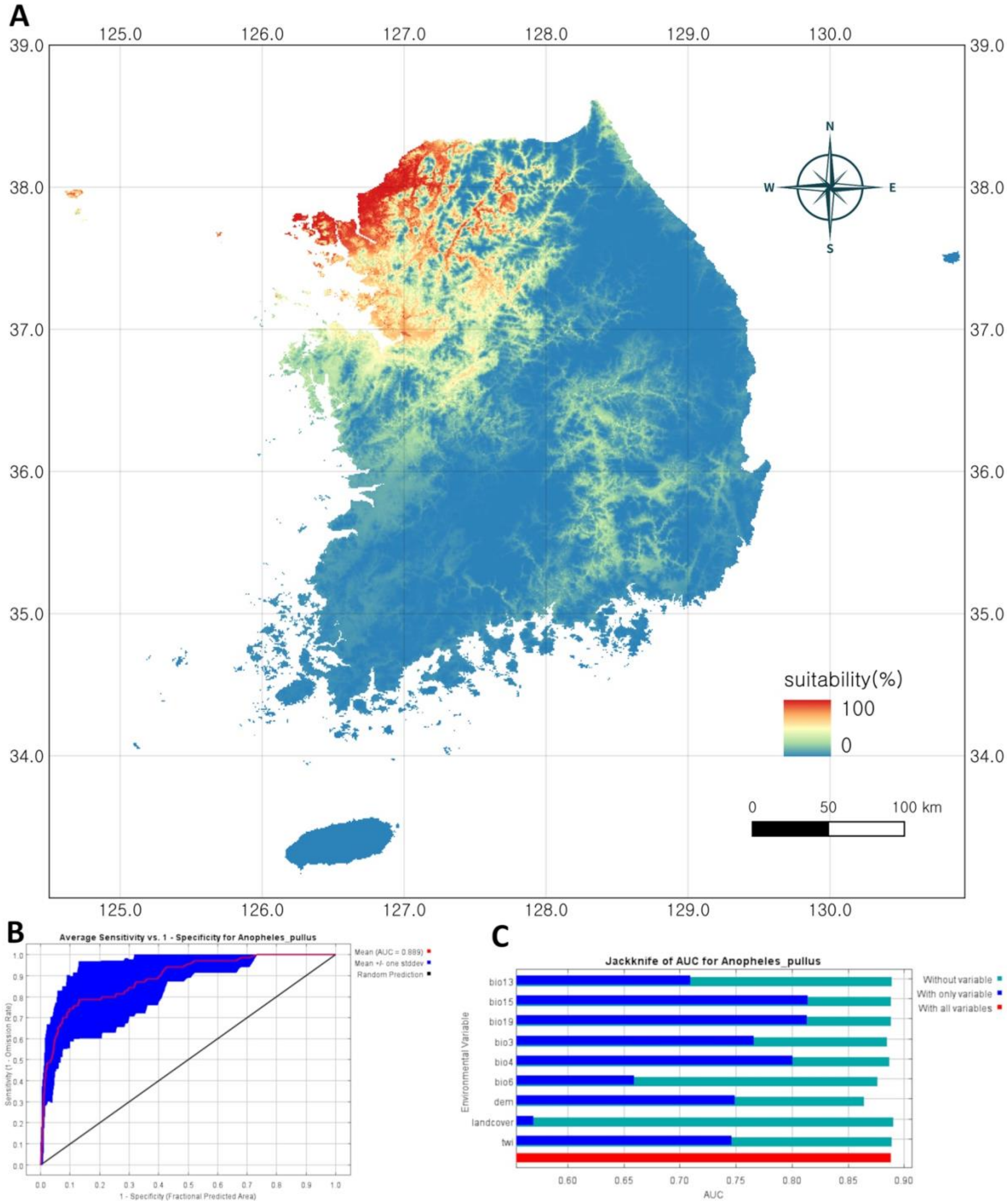

Figure S8. MaxEnt species distribution model results for Anopheles pullus. (A) Current predicted habitat suitability map for the Republic of Korea showing a northern-preferring distribution pattern. Warmer colors (red) indicate higher predicted suitability and cooler colors (blue) indicate lower suitability (range: 0-100%). (B) Receiver operating characteristic (ROC) curve showing model discrimination ability (mean AUC = 0.889). (C) Jackknife test of variable importance based on AUC. The final model used 9 environmental variables (Table S1) with optimal regularization multiplier = 2.5 and linear (L) feature class.

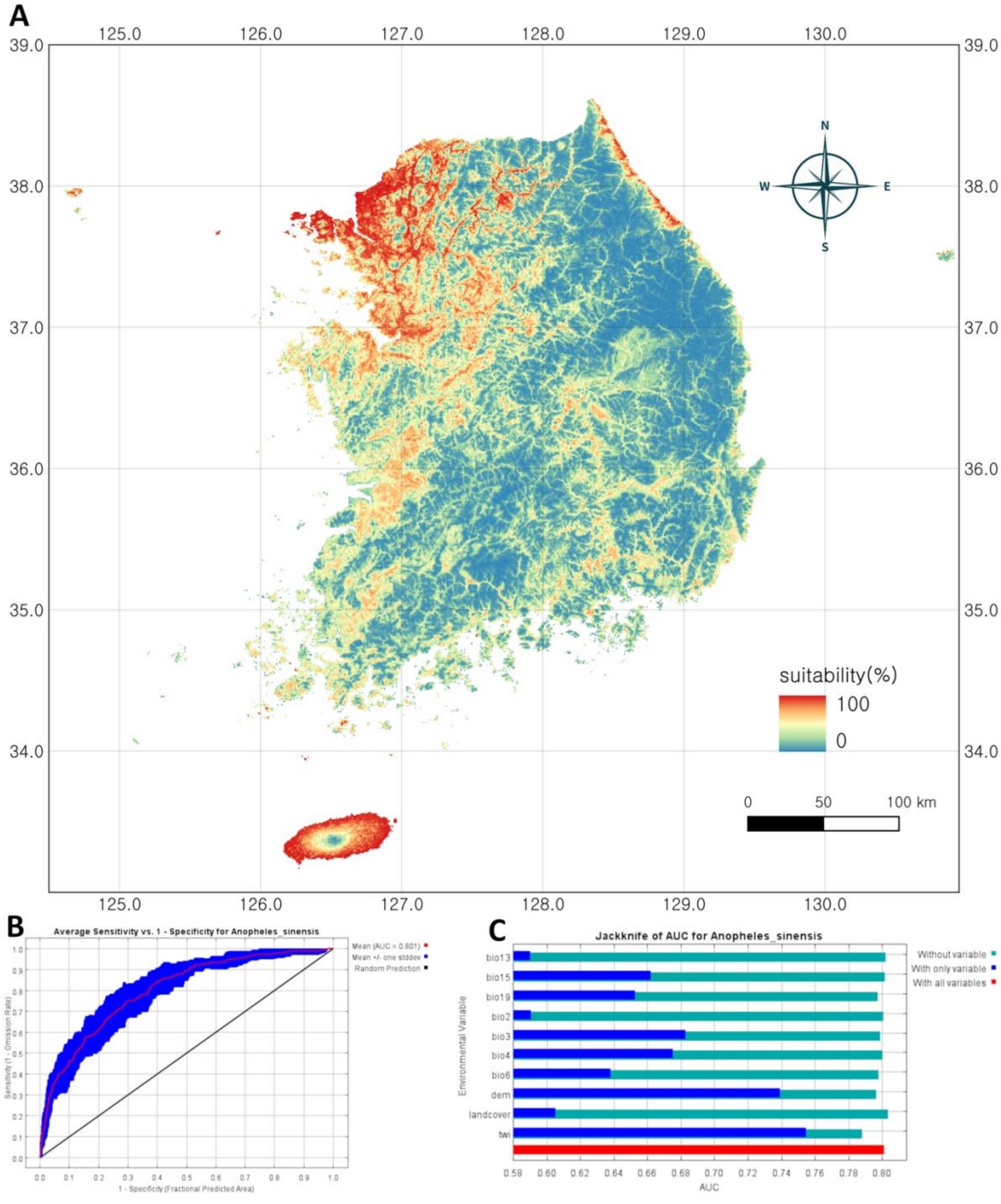

Figure S9. MaxEnt species distribution model results for Anopheles sinensis. (A) Current predicted habitat suitability map for the Republic of Korea. Warmer colors (red) indicate higher predicted suitability and cooler colors (blue) indicate lower suitability (range: 0-100%). (B) Receiver operating characteristic (ROC) curve showing model discrimination ability (mean AUC = 0.801). (C) Jackknife test of variable importance based on AUC. The final model used 10 environmental variables (Table S1) with optimal regularization multiplier = 3.0 and linear-quadratic-hinge (LQH) feature class.

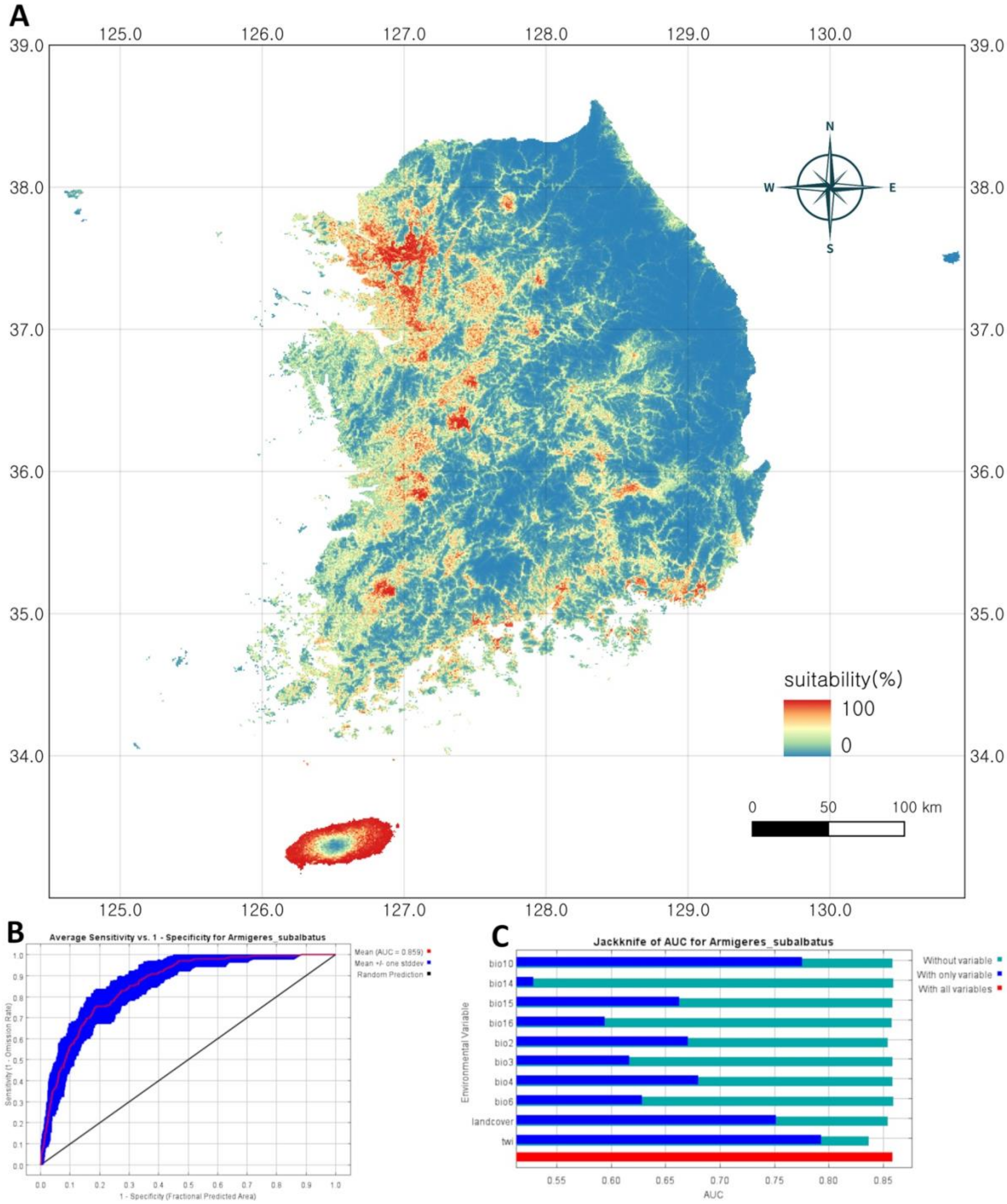

Figure S10. MaxEnt species distribution model results for Armigeres subalbatus. (A) Current predicted habitat suitability map for the Republic of Korea. Warmer colors (red) indicate higher predicted suitability and cooler colors (blue) indicate lower suitability (range: 0-100%). (B) Receiver operating characteristic (ROC) curve showing model discrimination ability (mean AUC = 0.859). (C) Jackknife test of variable importance based on AUC. The final model used 10 environmental variables (Table S1) with optimal regularization multiplier = 3.0 and linear-quadratic-hinge (LQH) feature class.

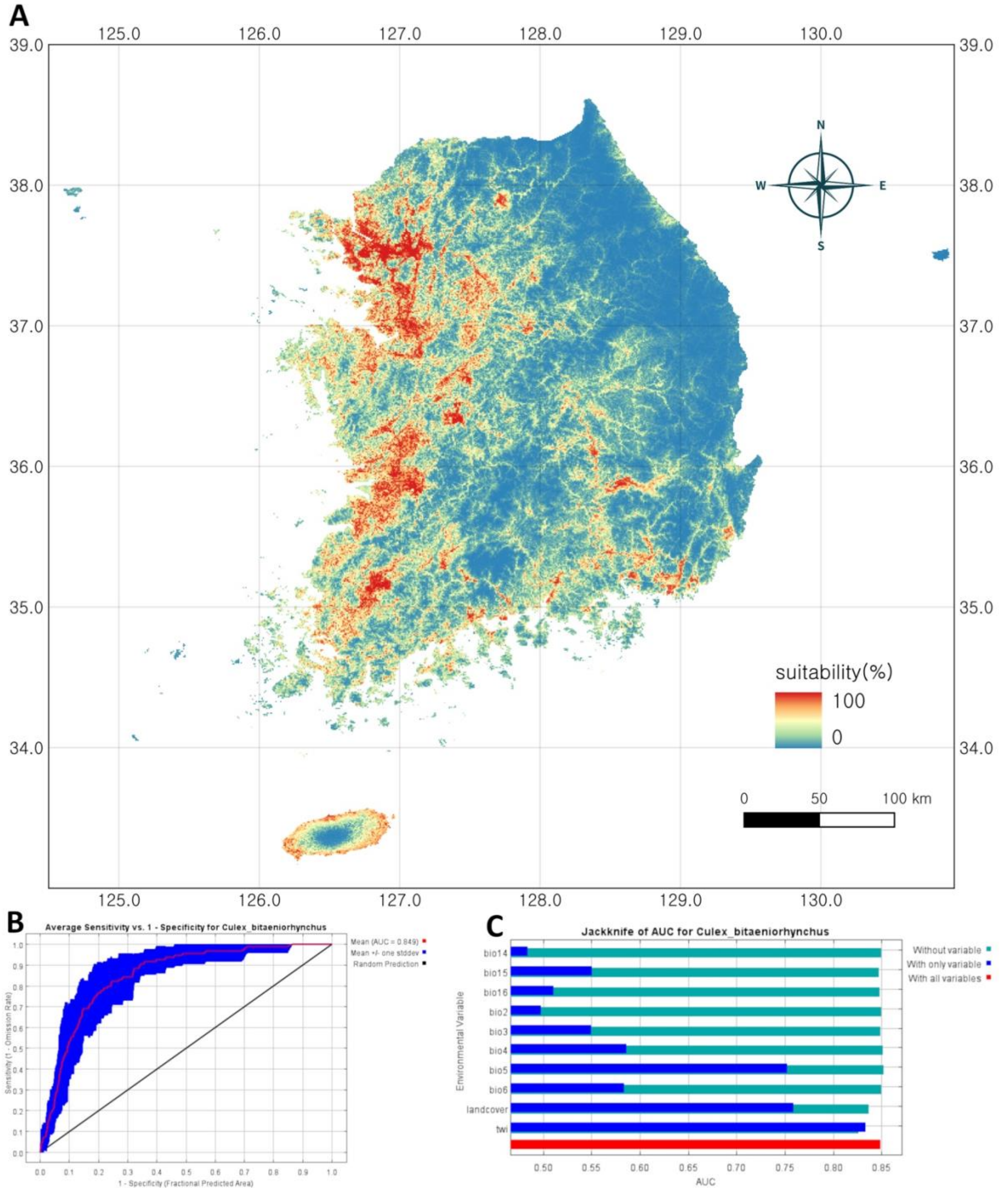

Figure S11. MaxEnt species distribution model results for Culex bitaeniorhynchus. (A) Current predicted habitat suitability map for the Republic of Korea. Warmer colors (red) indicate higher predicted suitability and cooler colors (blue) indicate lower suitability (range: 0-100%). (B) Receiver operating characteristic (ROC) curve showing model discrimination ability (mean AUC = 0.849). (C) Jackknife test of variable importance based on AUC. The final model used 10 environmental variables (Table S1) with optimal regularization multiplier = 3.5 and linear-quadratic (LQ) feature class.

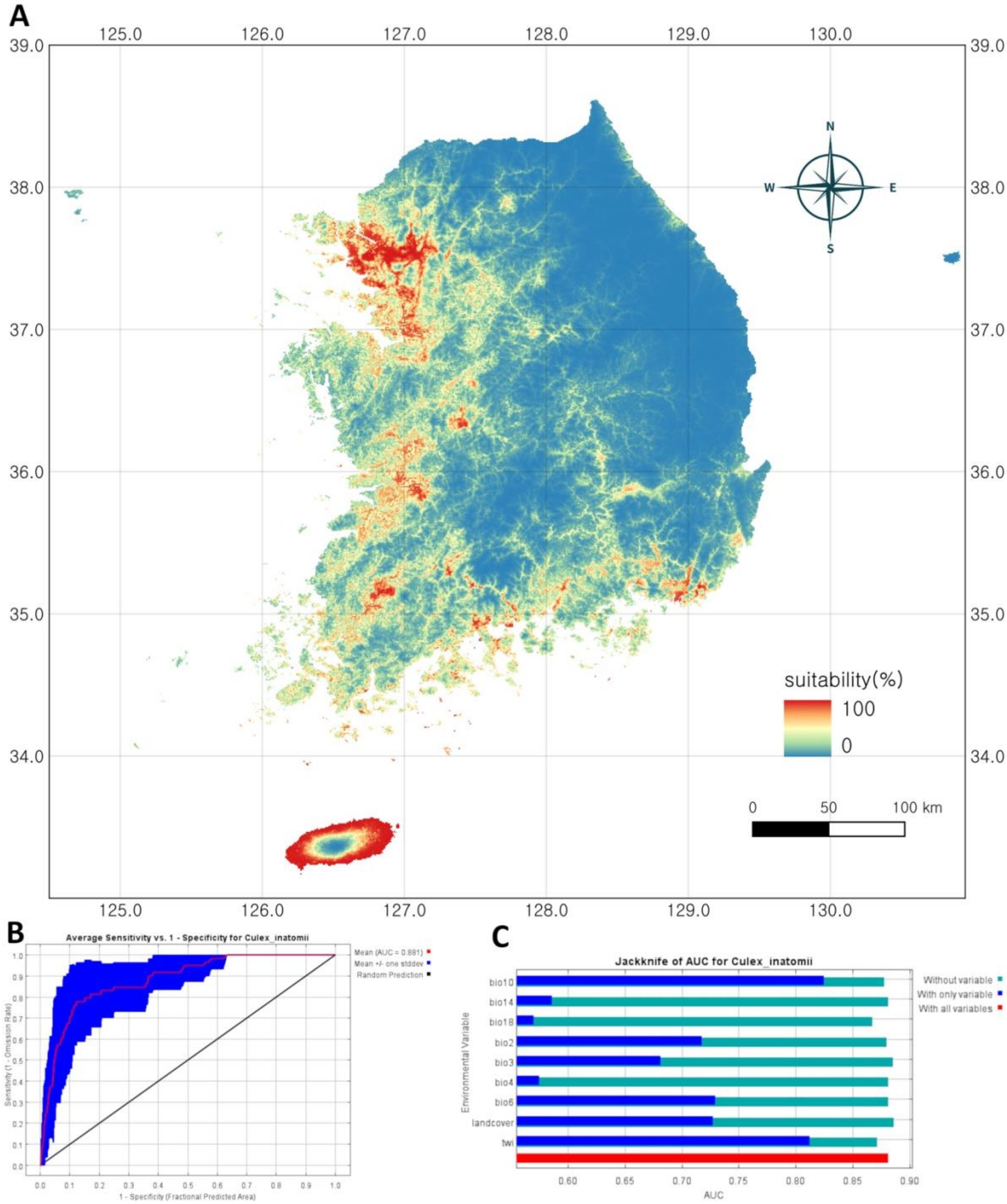

Figure S12. MaxEnt species distribution model results for Culex inatomii. (A) Current predicted habitat suitability map for the Republic of Korea showing a southwestern-preferring distribution pattern. Warmer colors (red) indicate higher predicted suitability and cooler colors (blue) indicate lower suitability (range: 0-100%). (B) Receiver operating characteristic (ROC) curve showing model discrimination ability (mean AUC = 0.881). (C) Jackknife test of variable importance based on AUC. The final model used 9 environmental variables (Table S1) with optimal regularization multiplier = 5.0 and linear-quadratic (LQ) feature class.

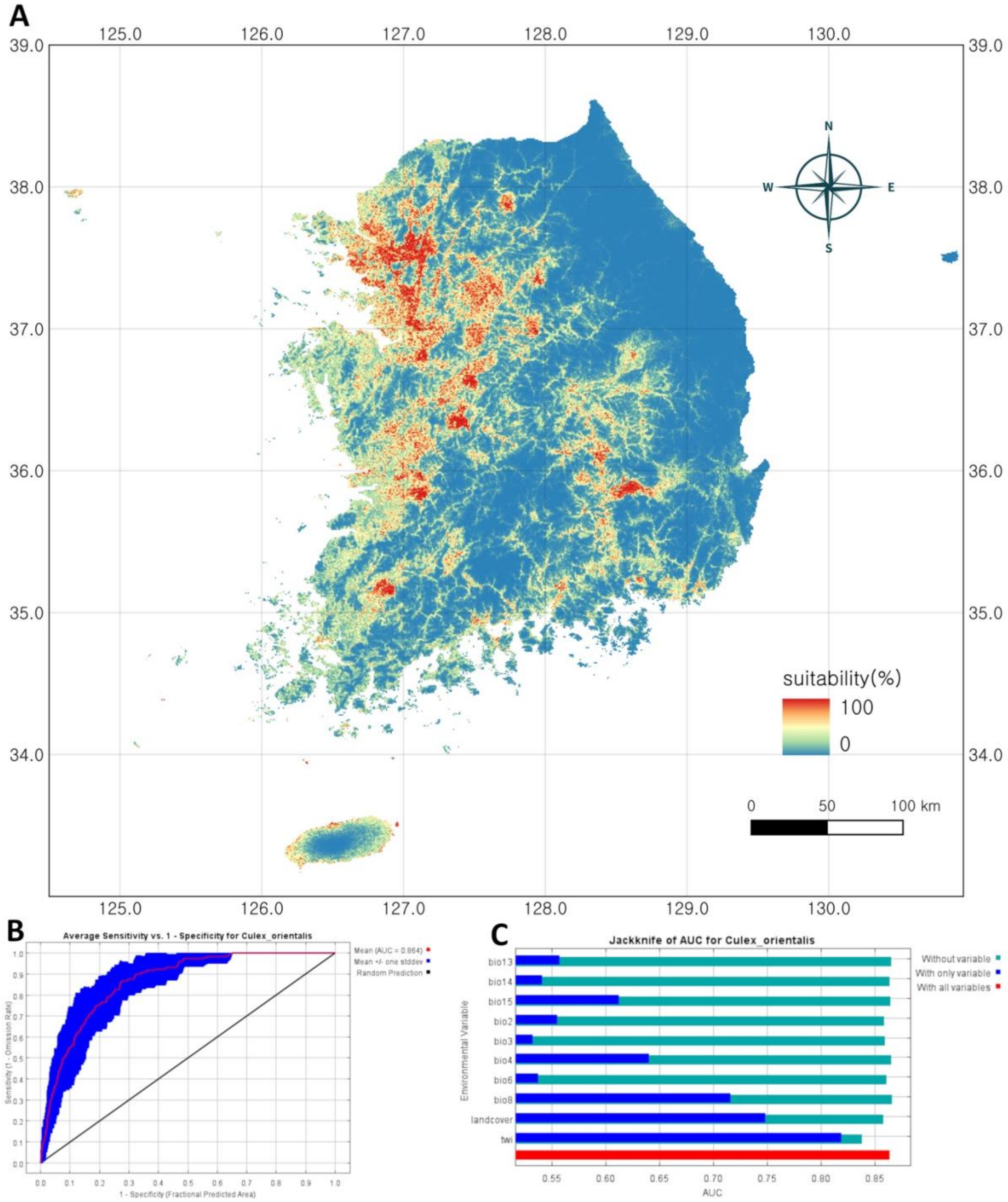

Figure S13. MaxEnt species distribution model results for Culex orientalis. (A) Current predicted habitat suitability map for the Republic of Korea. Warmer colors (red) indicate higher predicted suitability and cooler colors (blue) indicate lower suitability (range: 0-100%). (B) Receiver operating characteristic (ROC) curve showing model discrimination ability (mean AUC = 0.864). (C) Jackknife test of variable importance based on AUC. The final model used 10 environmental variables (Table S1) with optimal regularization multiplier = 1.0 and linear-quadratic (LQ) feature class.

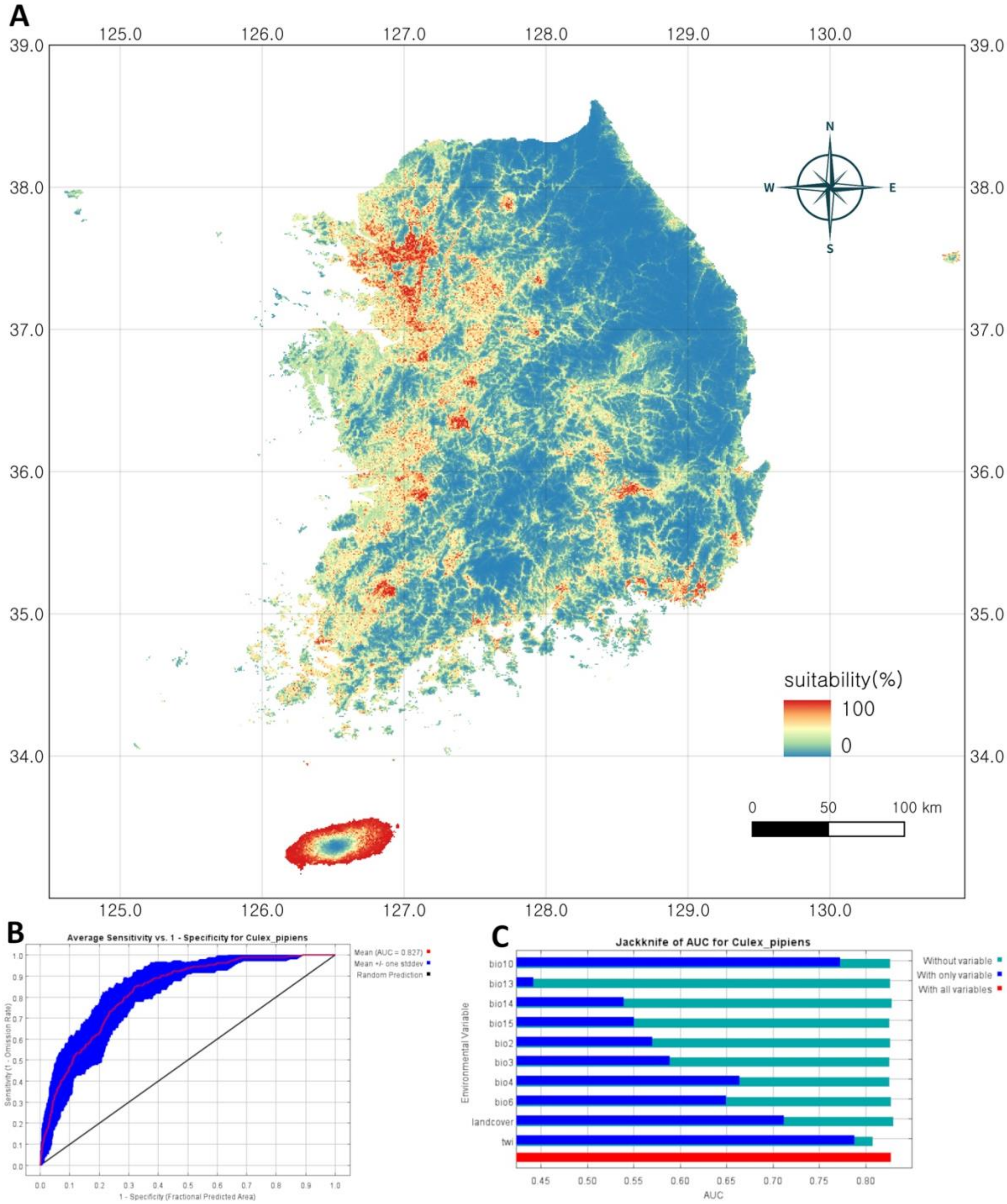

Figure S14. MaxEnt species distribution model results for Culex pipiens. (A) Current predicted habitat suitability map for the Republic of Korea. Warmer colors (red) indicate higher predicted suitability and cooler colors (blue) indicate lower suitability (range: 0-100%). (B) Receiver operating characteristic (ROC) curve showing model discrimination ability (mean AUC = 0.827). (C) Jackknife test of variable importance based on AUC. The final model used 10 environmental variables (Table S1) with optimal regularization multiplier = 1.0 and linear-quadratic (LQ) feature class.

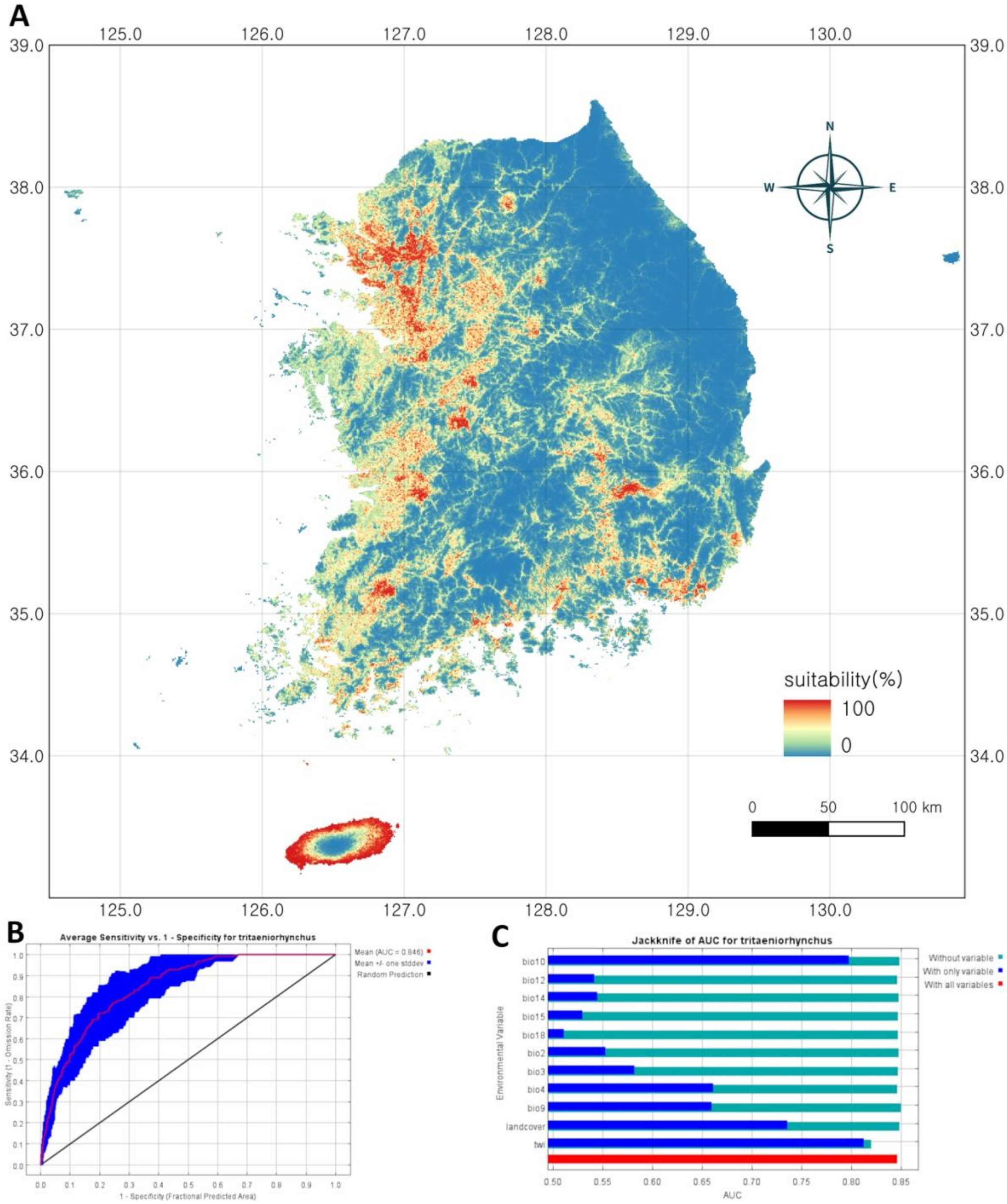

Figure S15. MaxEnt species distribution model results for Culex tritaeniorhynchus. (A) Current predicted habitat suitability map for the Republic of Korea. Warmer colors (red) indicate higher predicted suitability and cooler colors (blue) indicate lower suitability (range: 0-100%). (B) Receiver operating characteristic (ROC) curve showing model discrimination ability (mean AUC = 0.846). (C) Jackknife test of variable importance based on AUC. The final model used 11 environmental variables (Table S1) with optimal regularization multiplier = 1.5 and linear-quadratic (LQ) feature class.

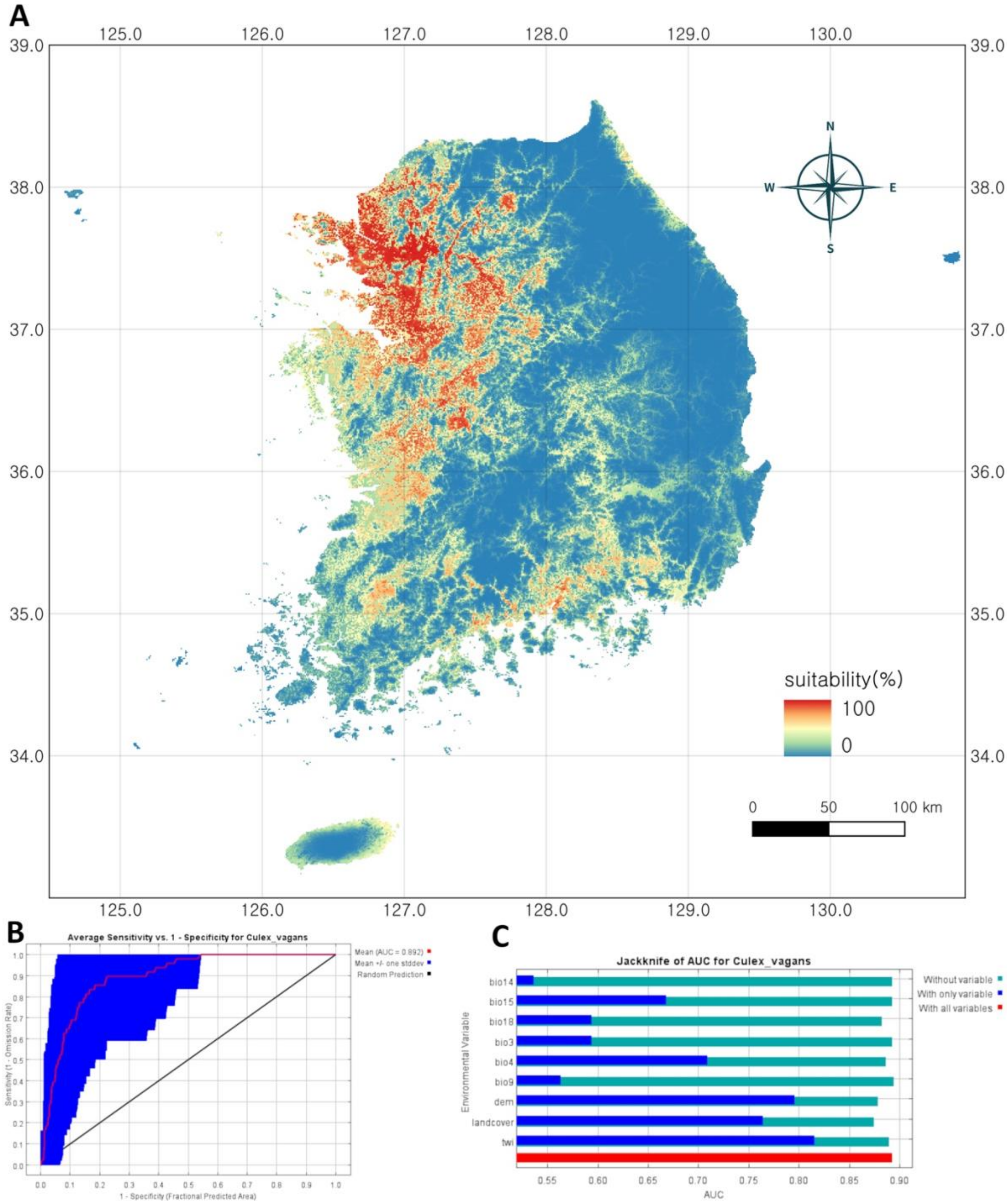

Figure S16. MaxEnt species distribution model results for Culex vagans. (A) Current predicted habitat suitability map for the Republic of Korea. Warmer colors (red) indicate higher predicted suitability and cooler colors (blue) indicate lower suitability (range: 0-100%). (B) Receiver operating characteristic (ROC) curve showing model discrimination ability (mean AUC = 0.892). (C) Jackknife test of variable importance based on AUC. The final model used 9 environmental variables (Table S1) with optimal regularization multiplier = 4.5 and linear-quadratic-hinge (LQH) feature class.

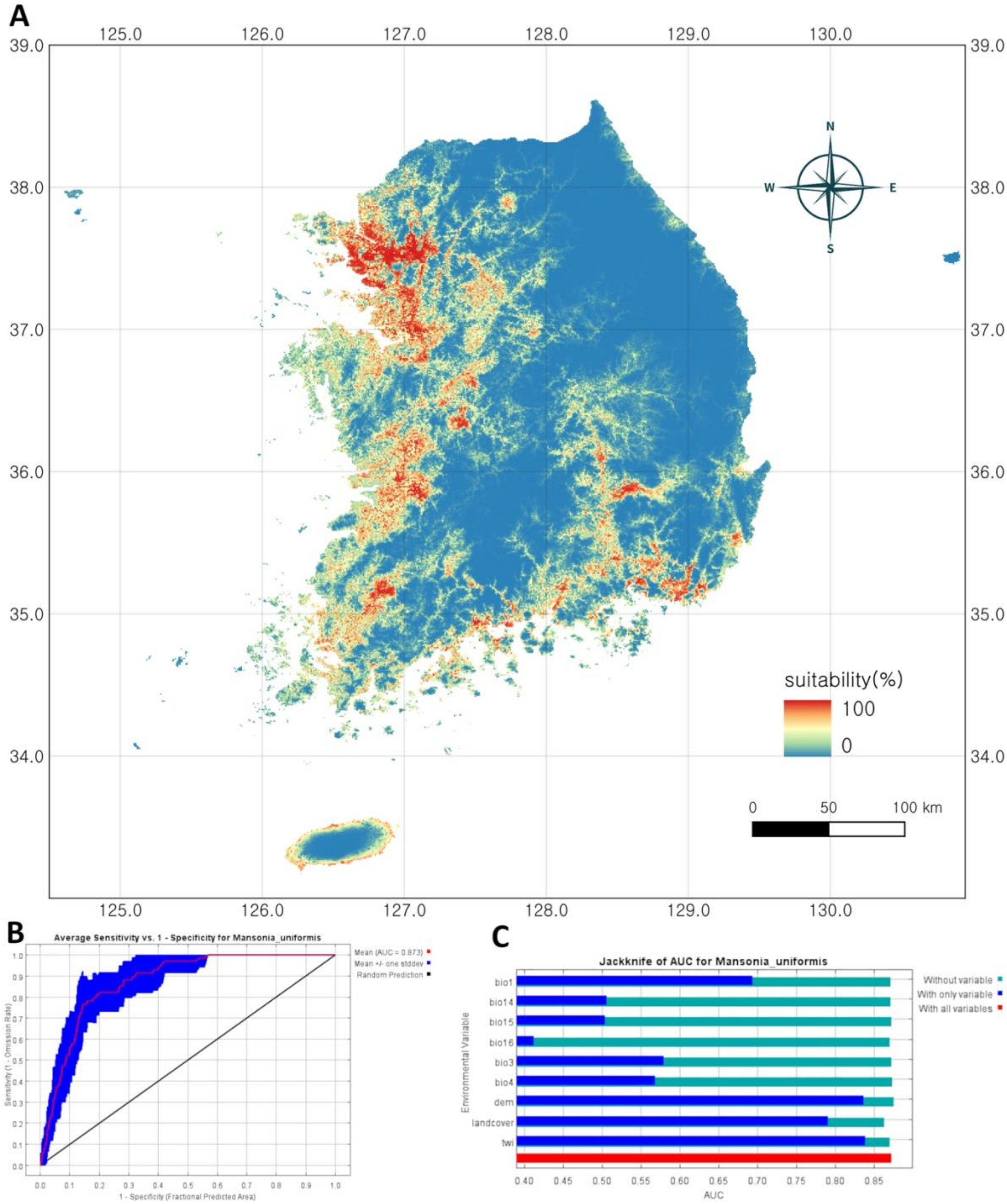

Figure S17. MaxEnt species distribution model results for Mansonia uniformis. (A) Current predicted habitat suitability map for the Republic of Korea showing a southwestern-preferring distribution pattern. Warmer colors (red) indicate higher predicted suitability and cooler colors (blue) indicate lower suitability (range: 0-100%). (B) Receiver operating characteristic (ROC) curve showing model discrimination ability (mean AUC = 0.873). (C) Jackknife test of variable importance based on AUC. The final model used 9 environmental variables (Table S1) with optimal regularization multiplier = 1.5 and linear-quadratic (LQ) feature class.

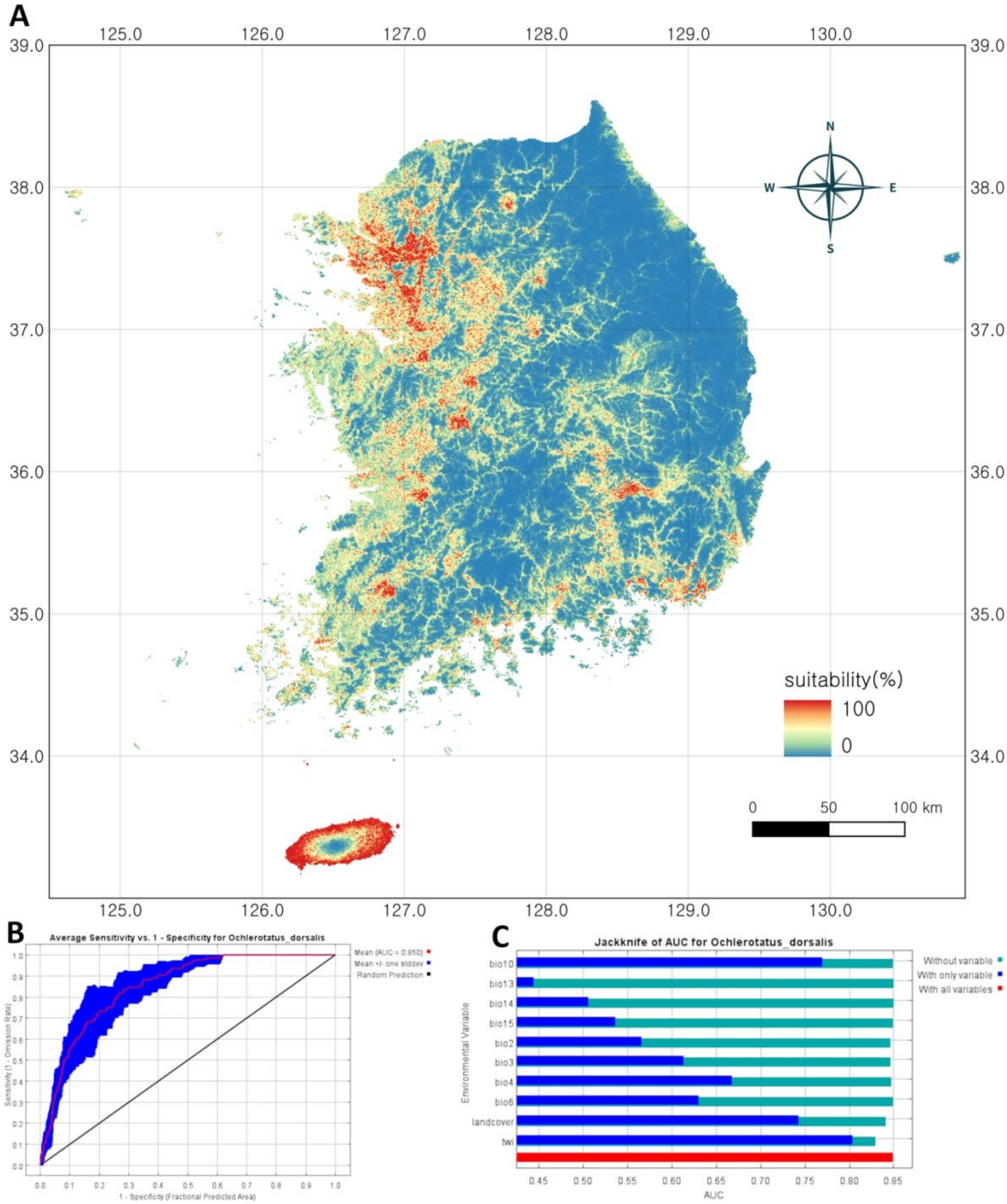

Figure S18. MaxEnt species distribution model results for Ochlerotatus dorsalis. (A) Current predicted habitat suitability map for the Republic of Korea. Warmer colors (red) indicate higher predicted suitability and cooler colors (blue) indicate lower suitability (range: 0-100%). (B) Receiver operating characteristic (ROC) curve showing model discrimination ability (mean AUC = 0.850). (C) Jackknife test of variable importance based on AUC. The final model used 10 environmental variables (Table S1) with optimal regularization multiplier = 1.0 and linear-quadratic (LQ) feature class.

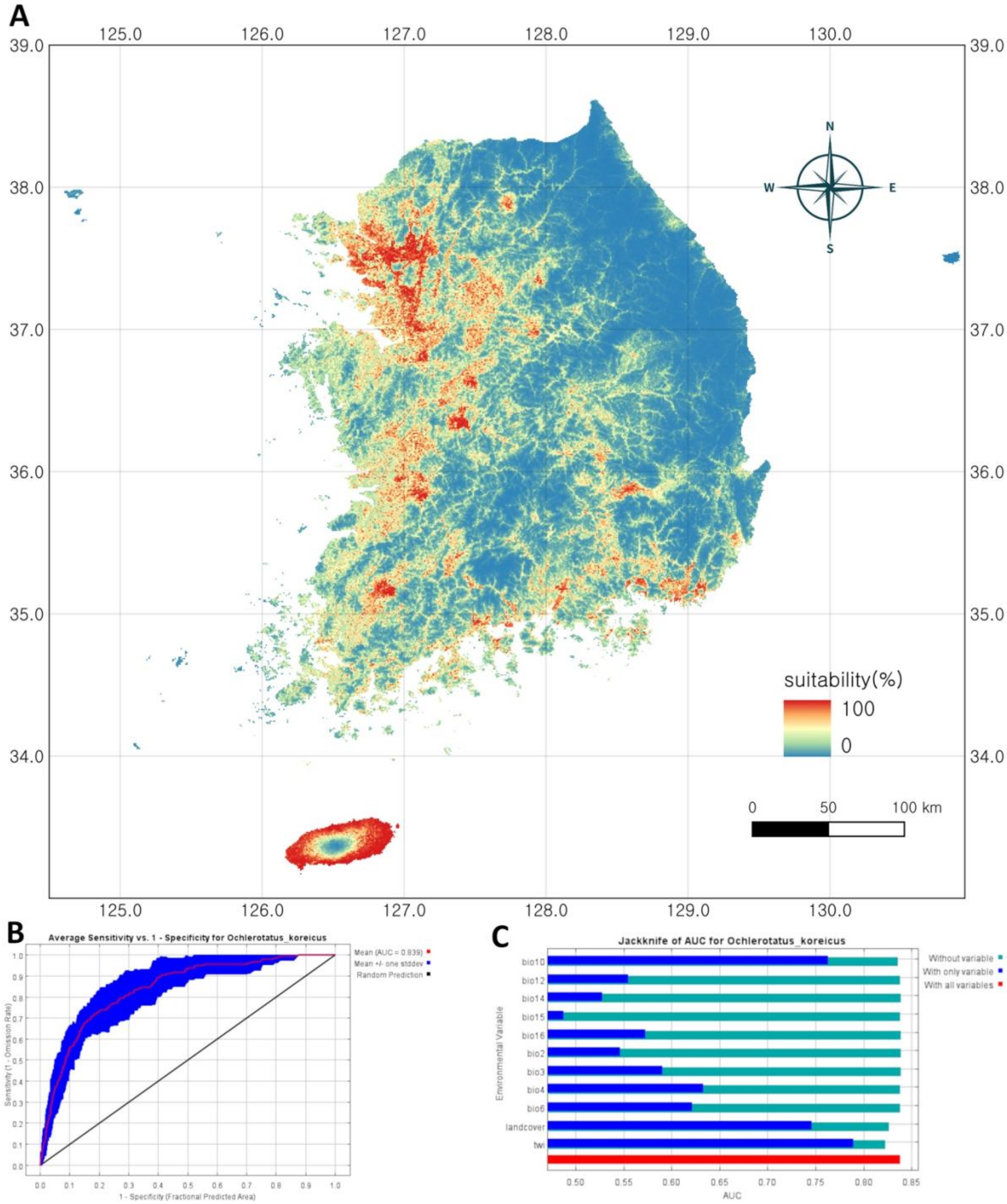

Figure S19. MaxEnt species distribution model results for Ochlerotatus koreicus. (A) Current predicted habitat suitability map for the Republic of Korea. Warmer colors (red) indicate higher predicted suitability and cooler colors (blue) indicate lower suitability (range: 0-100%). (B) Receiver operating characteristic (ROC) curve showing model discrimination ability (mean AUC = 0.839). (C) Jackknife test of variable importance based on AUC. The final model used 11 environmental variables (Table S1) with optimal regularization multiplier = 2.5 and linear-quadratic (LQ) feature class.

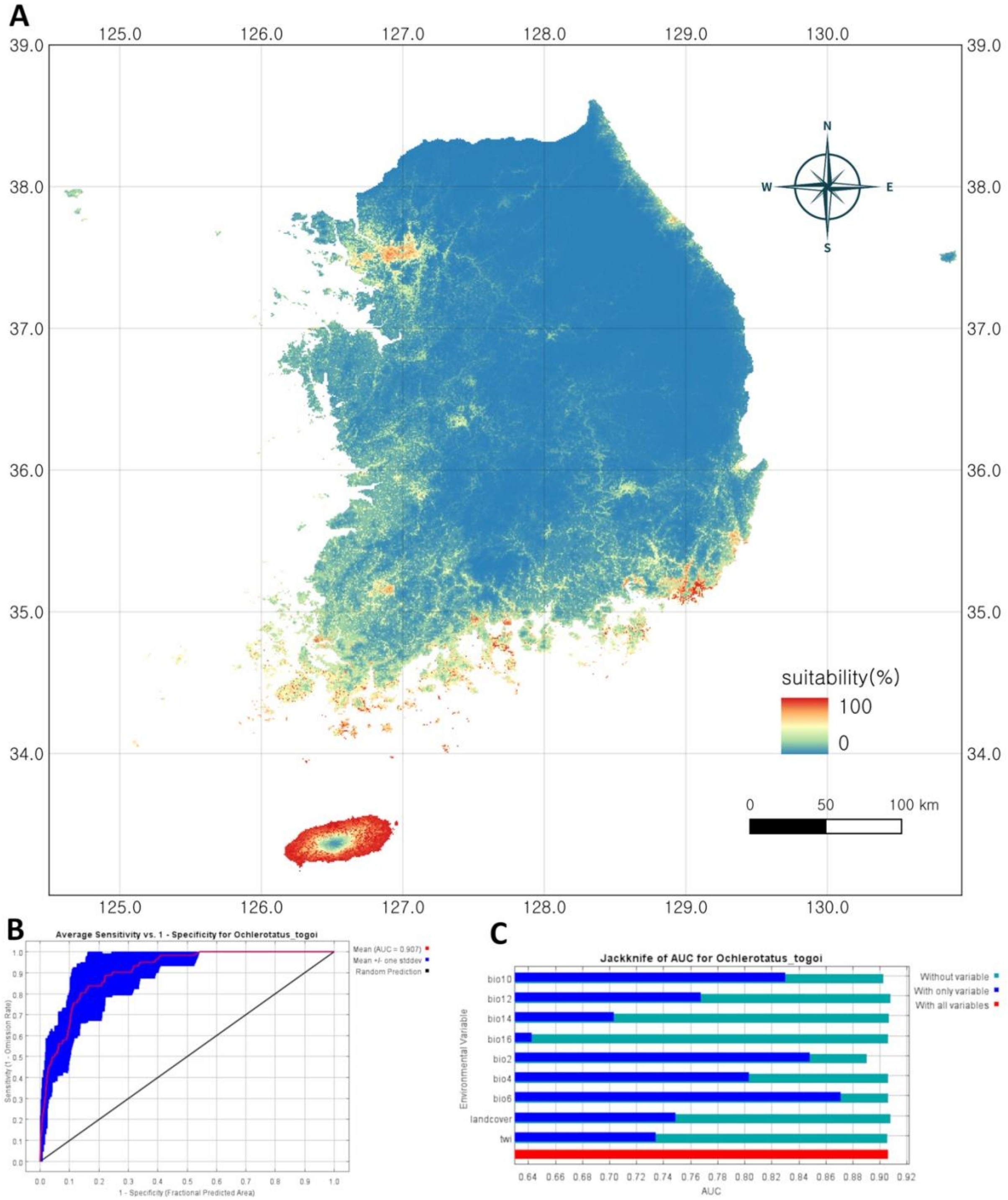

Figure S20. MaxEnt species distribution model results for Ochlerotatus togoi. (A) Current predicted habitat suitability map for the Republic of Korea showing a southern-preferring distribution pattern. Warmer colors (red) indicate higher predicted suitability and cooler colors (blue) indicate lower suitability (range: 0-100%). (B) Receiver operating characteristic (ROC) curve showing model discrimination ability (mean AUC = 0.907). (C) Jackknife test of variable importance based on AUC. The final model used 9 environmental variables (Table S1) with optimal regularization multiplier = 1.5 and linear-quadratic (LQ) feature class.

Response Curves of Key Environmental Variables by Species

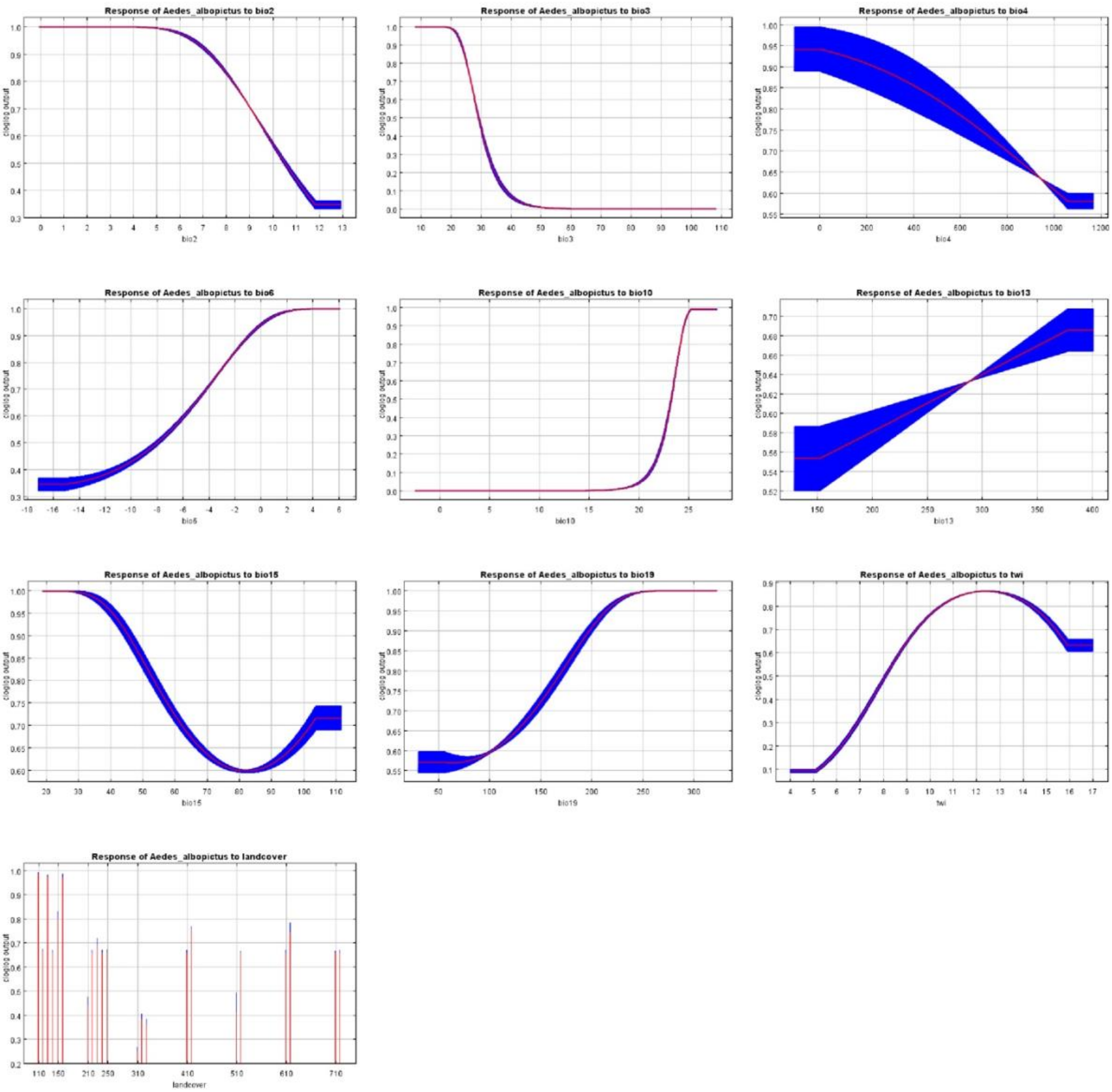

Figure S21. Response curves of Aedes albopictus to environmental variables retained in the final MaxEnt model. Each panel shows the predicted habitat suitability (y-axis, 0-1) as a function of a single environmental variable (x-axis), with all other variables held at their mean values. The blue line and shading represent the mean and standard deviation across replicate model runs. Variables shown correspond to the species-specific variable set listed in Table S1.

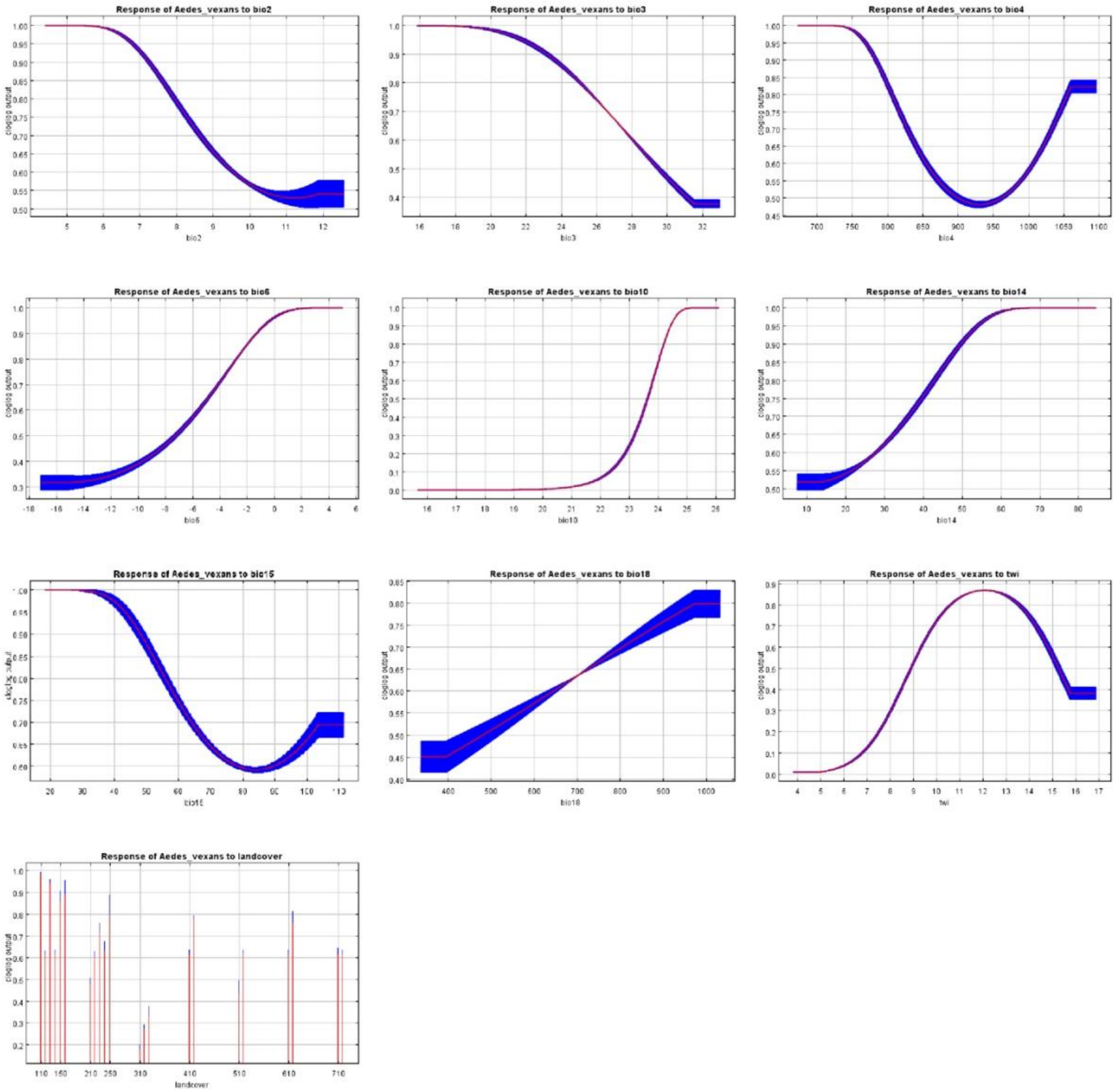

Figure S22. Response curves of Aedes vexans to environmental variables retained in the final MaxEnt model. Each panel shows predicted habitat suitability as a function of a single variable, with all other variables held at their mean values. See Table S1 for the variable set.

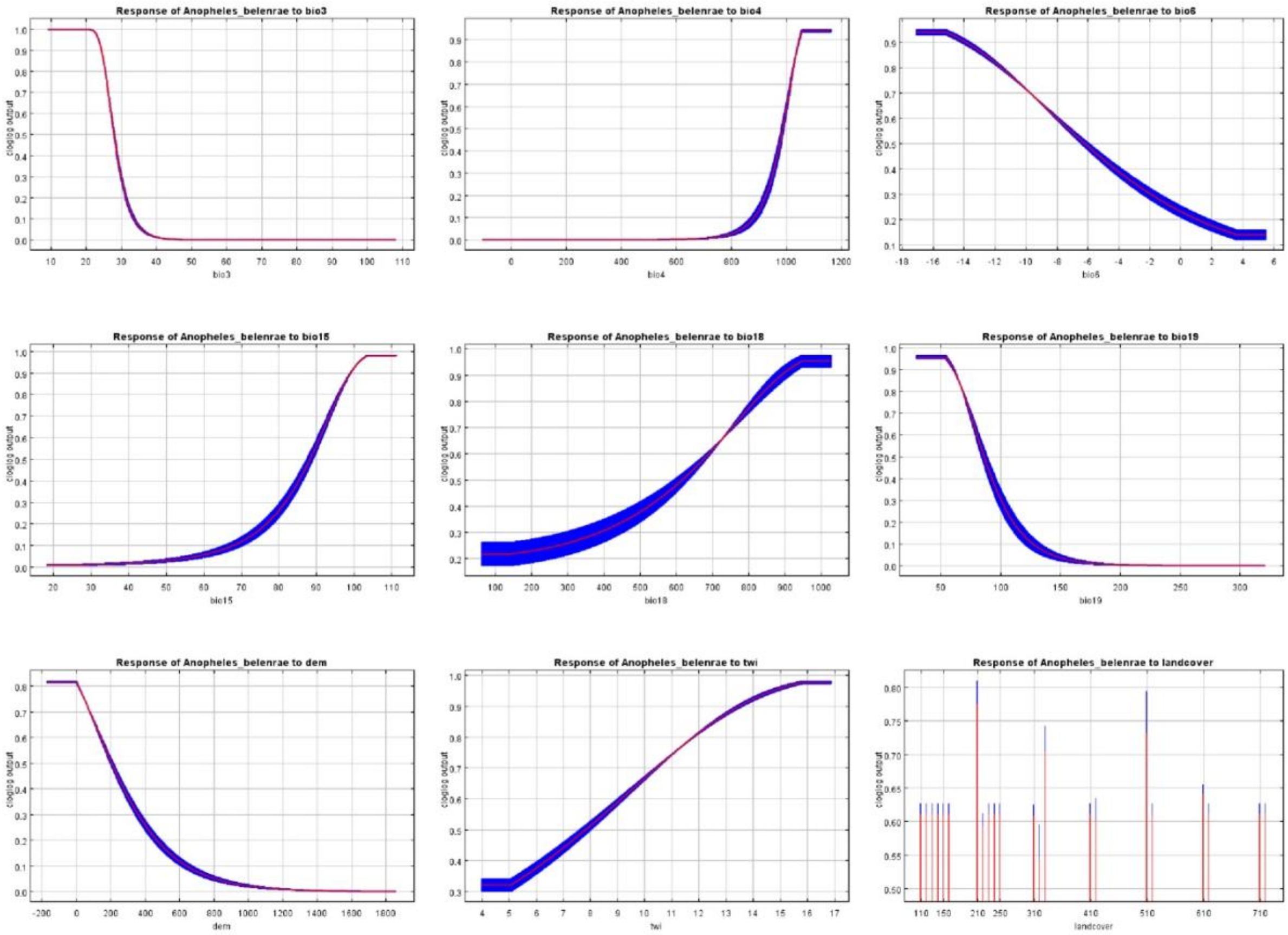

Figure S23. Response curves of Anopheles belenrae to environmental variables retained in the final MaxEnt model. Each panel shows predicted habitat suitability as a function of a single variable, with all other variables held at their mean values. See Table S1 for the variable set.

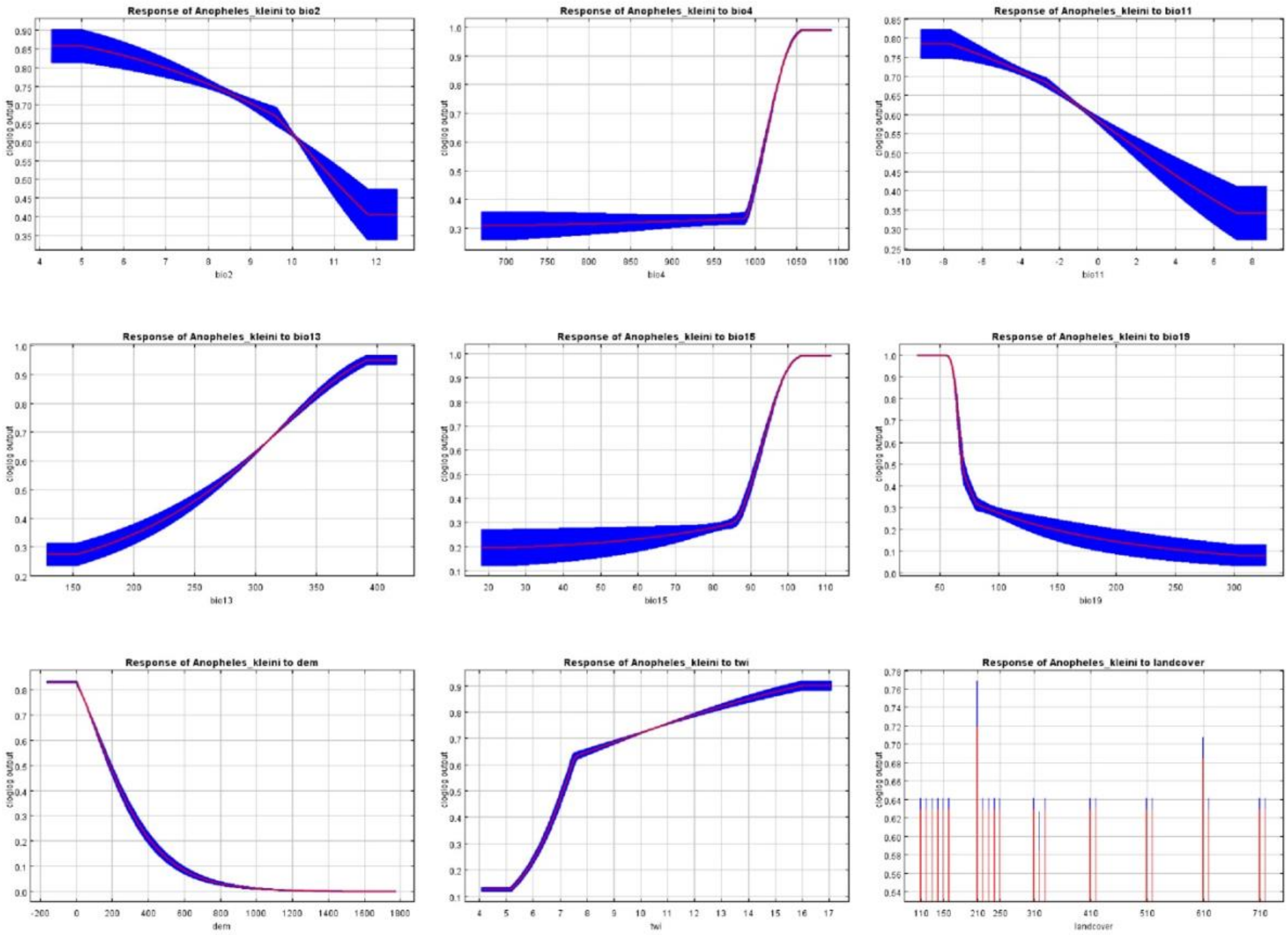

Figure S24. Response curves of Anopheles kleini to environmental variables retained in the final MaxEnt model. Each panel shows predicted habitat suitability as a function of a single variable, with all other variables held at their mean values. See Table S1 for the variable set.

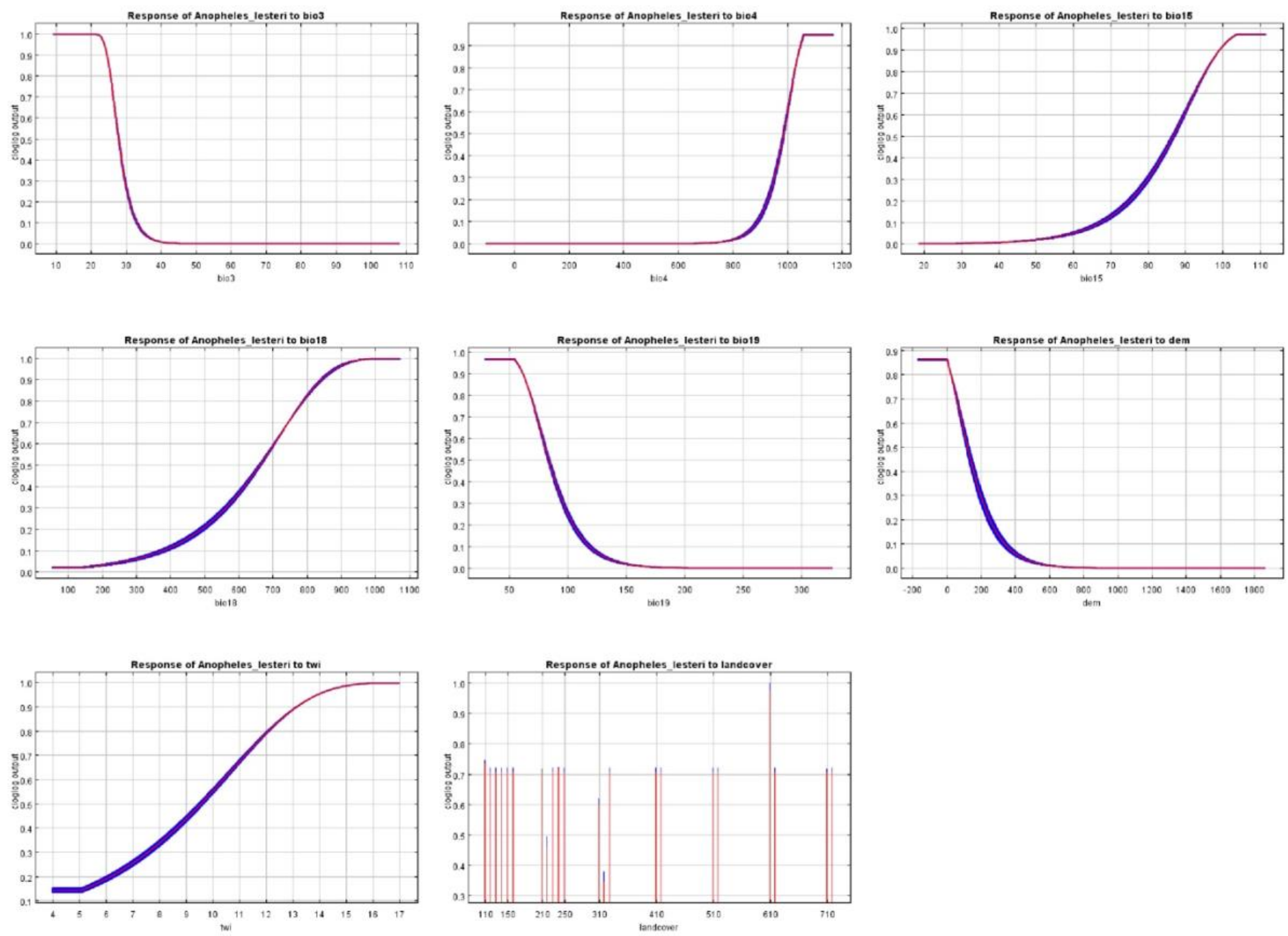

Figure S25. Response curves of Anopheles lesteri to environmental variables retained in the final MaxEnt model. Each panel shows predicted habitat suitability as a function of a single variable, with all other variables held at their mean values. See Table S1 for the variable set.

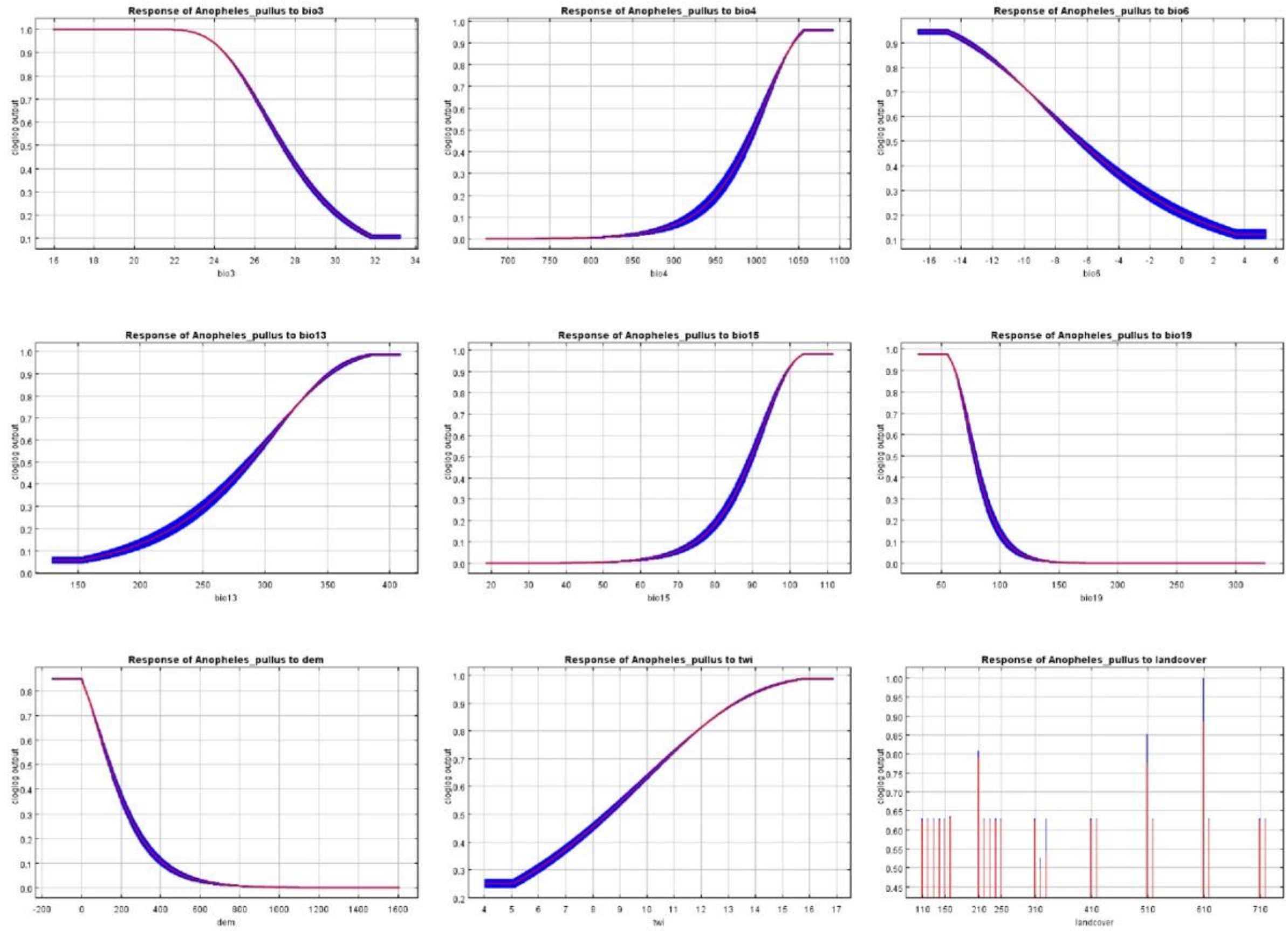

Figure S26. Response curves of Anopheles pullus to environmental variables retained in the final MaxEnt model. Each panel shows predicted habitat suitability as a function of a single variable, with all other variables held at their mean values. See Table S1 for the variable set.

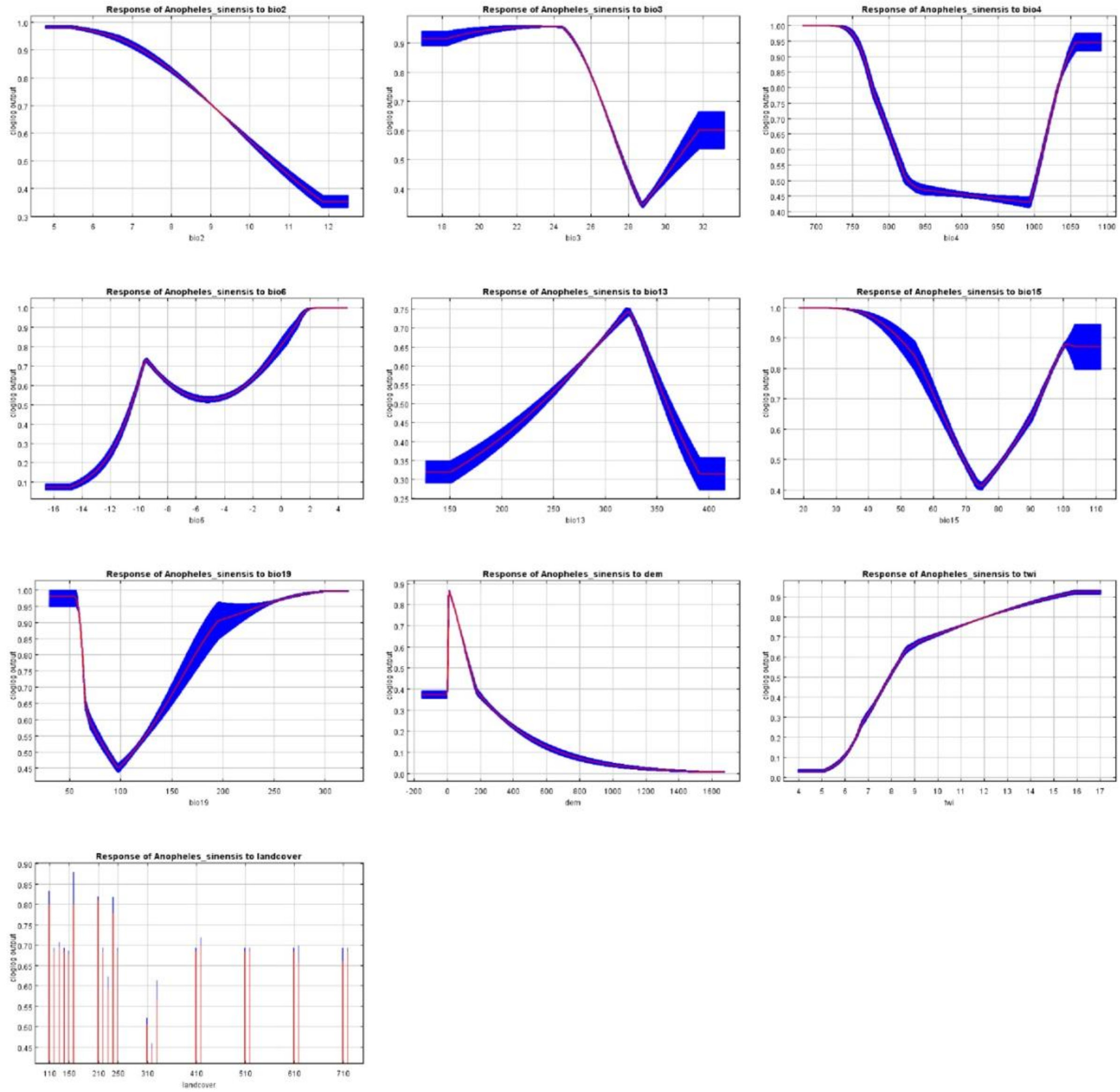

Figure S27. Response curves of Anopheles sinensis to environmental variables retained in the final MaxEnt model. Each panel shows predicted habitat suitability as a function of a single variable, with all other variables held at their mean values. See Table S1 for the variable set.

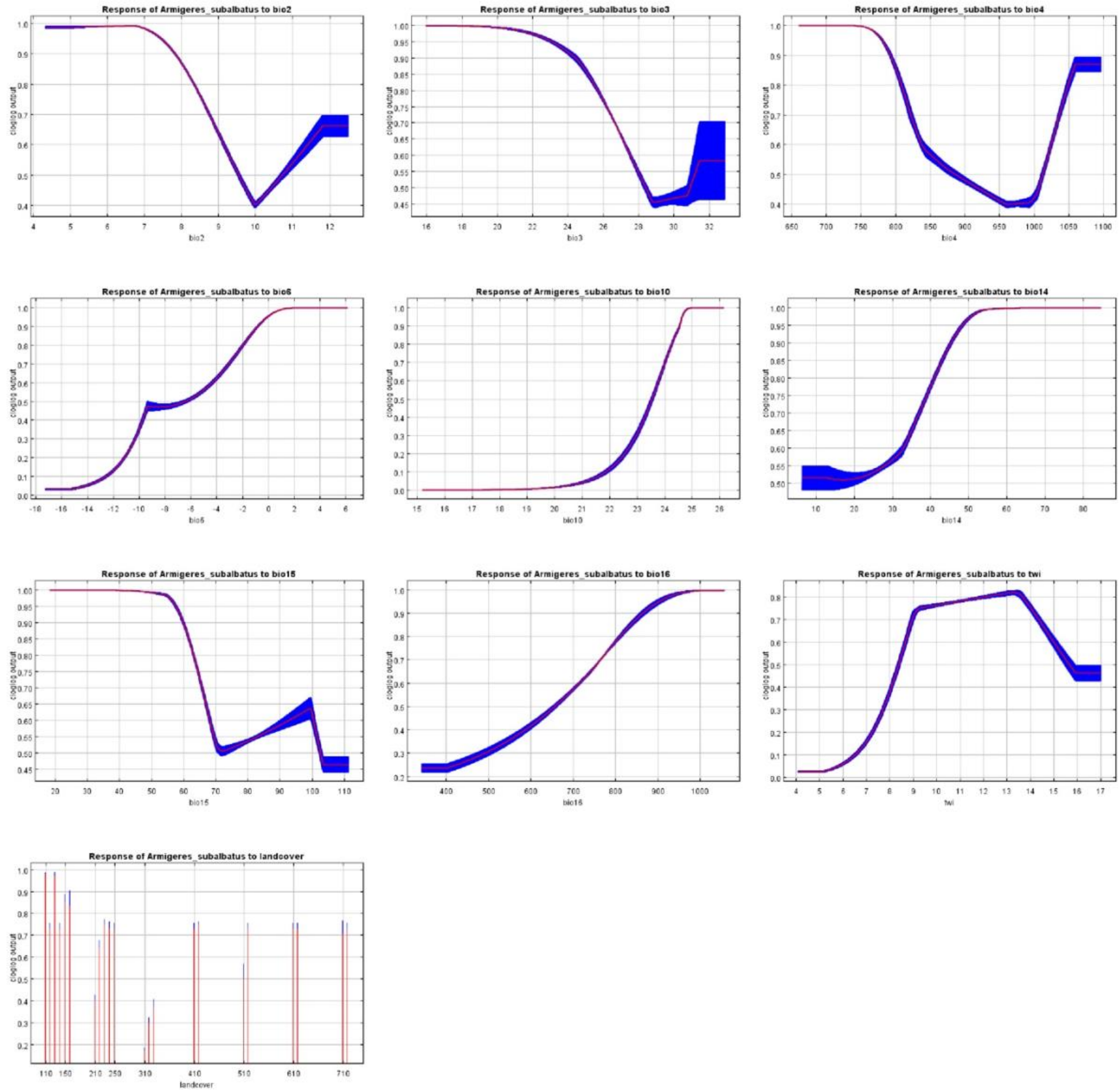

Figure S28. Response curves of Armigeres subalbatus to environmental variables retained in the final MaxEnt model. Each panel shows predicted habitat suitability as a function of a single variable, with all other variables held at their mean values. See Table S1 for the variable set.

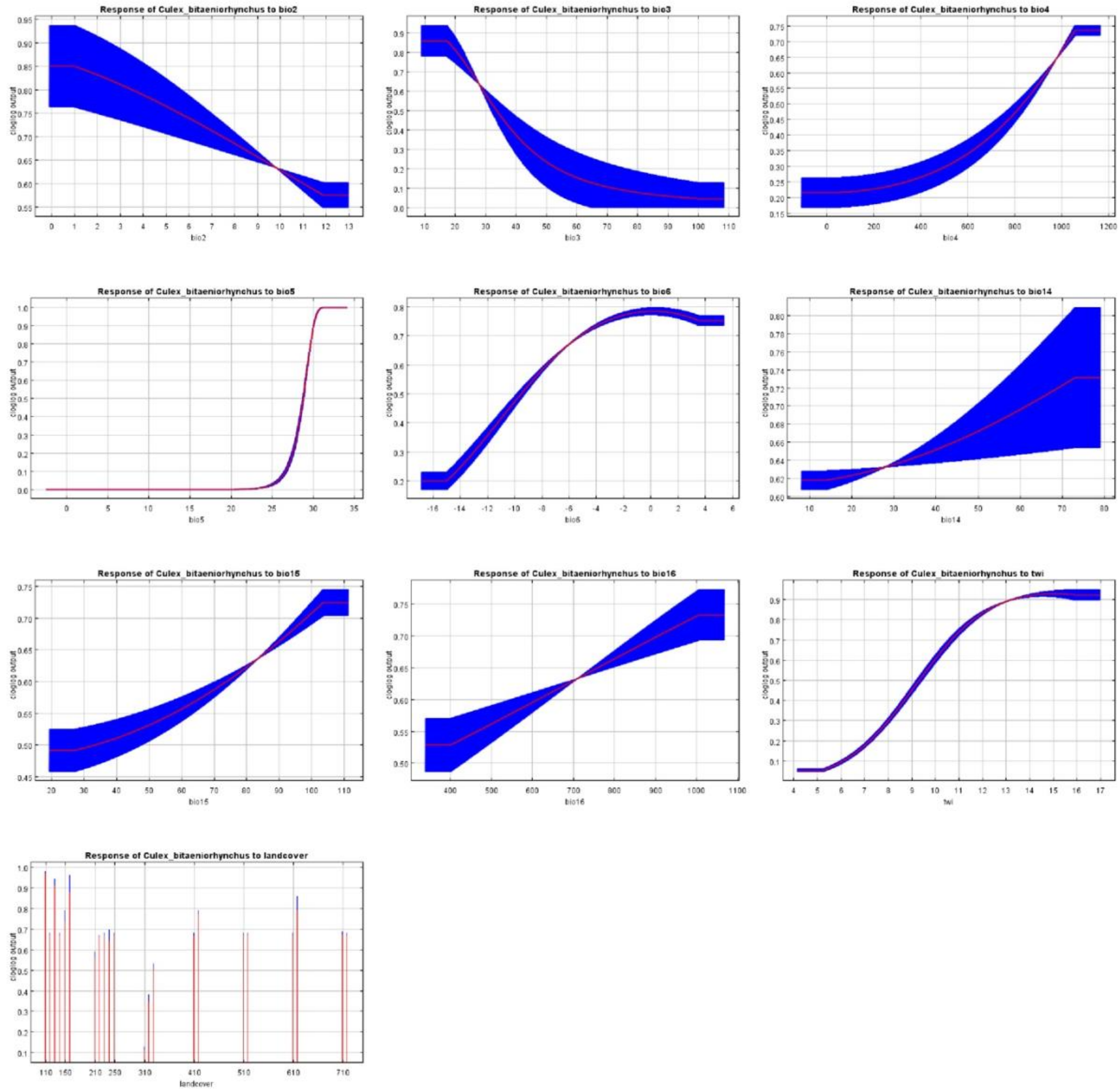

Figure S29. Response curves of Culex bitaeniorhynchus to environmental variables retained in the final MaxEnt model. Each panel shows predicted habitat suitability as a function of a single variable, with all other variables held at their mean values. See Table S1 for the variable set.

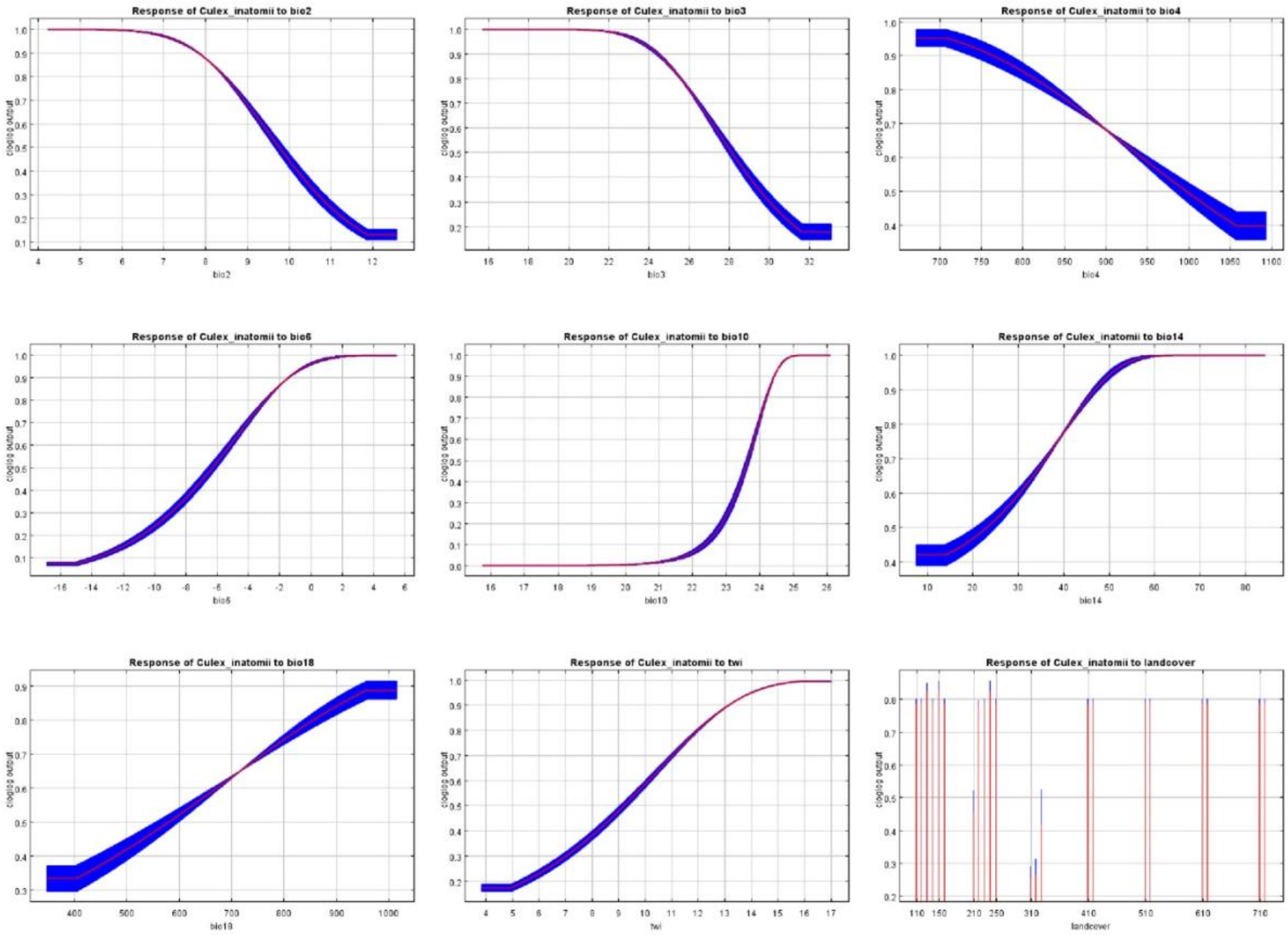

Figure S30. Response curves of Culex inatomii to environmental variables retained in the final MaxEnt model. Each panel shows predicted habitat suitability as a function of a single variable, with all other variables held at their mean values. See Table S1 for the variable set.

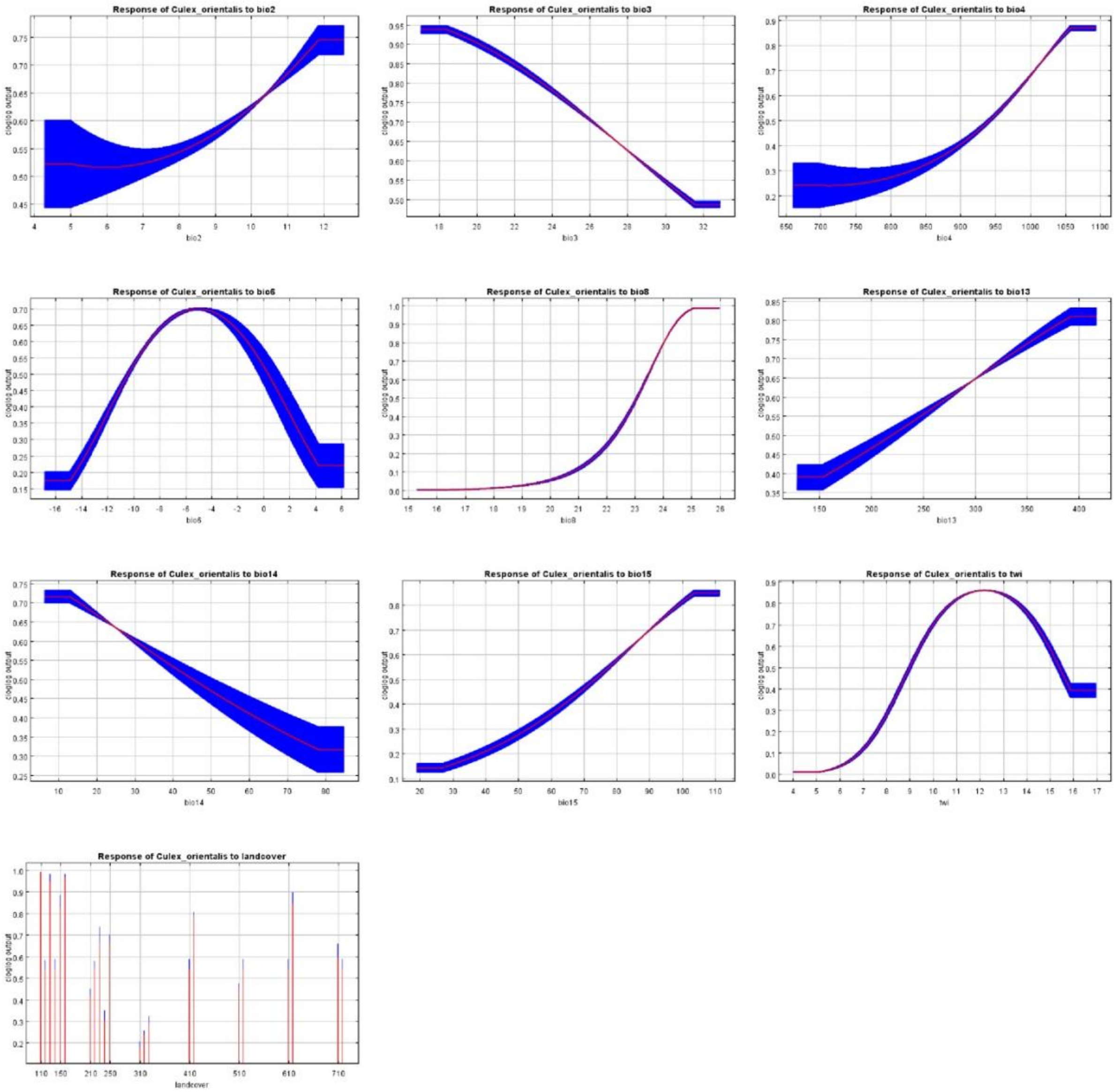

Figure S31. Response curves of Culex orientalis to environmental variables retained in the final MaxEnt model. Each panel shows predicted habitat suitability as a function of a single variable, with all other variables held at their mean values. See Table S1 for the variable set.

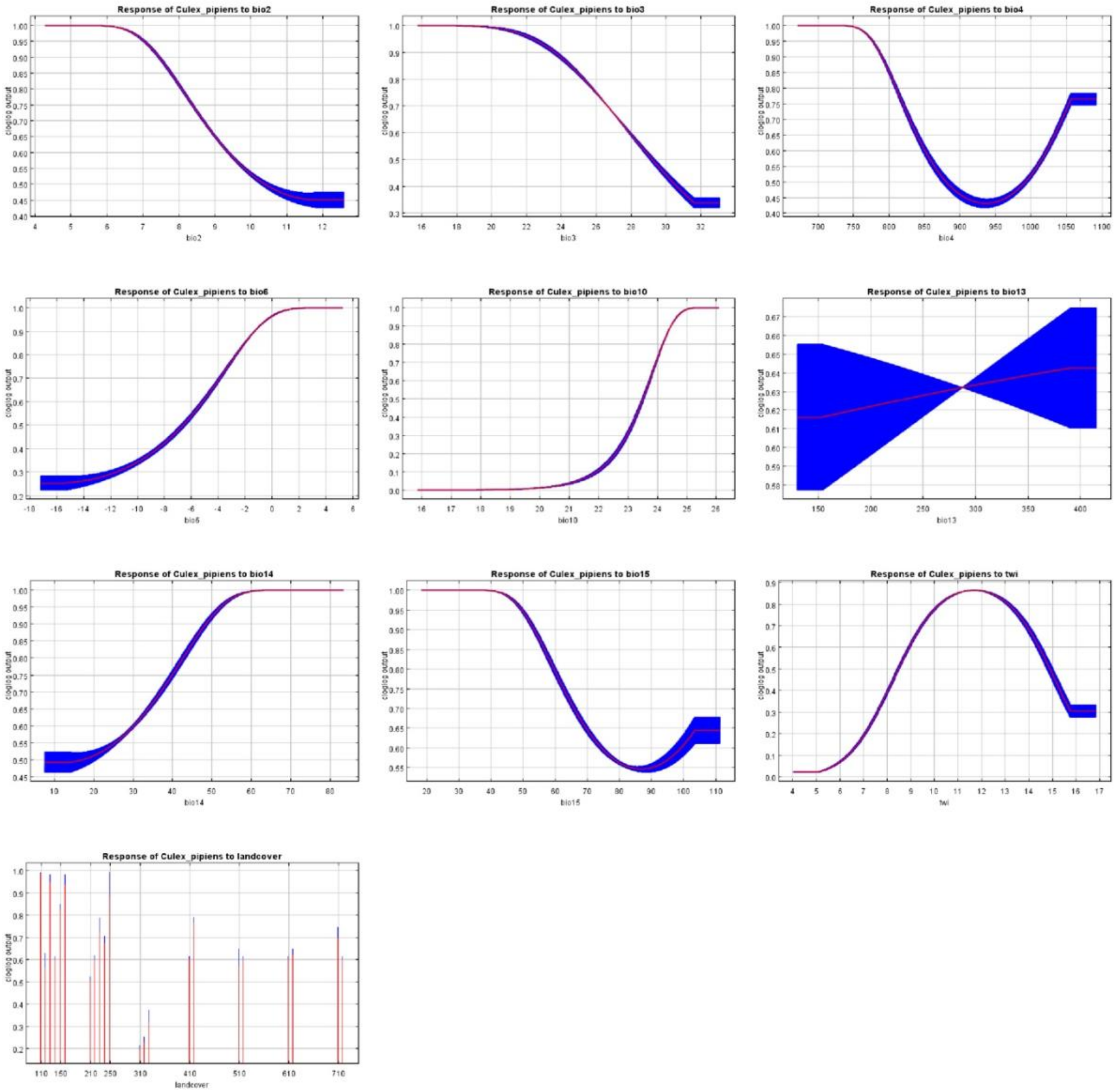

Figure S32. Response curves of Culex pipiens to environmental variables retained in the final MaxEnt model. Each panel shows predicted habitat suitability as a function of a single variable, with all other variables held at their mean values. See Table S1 for the variable set.

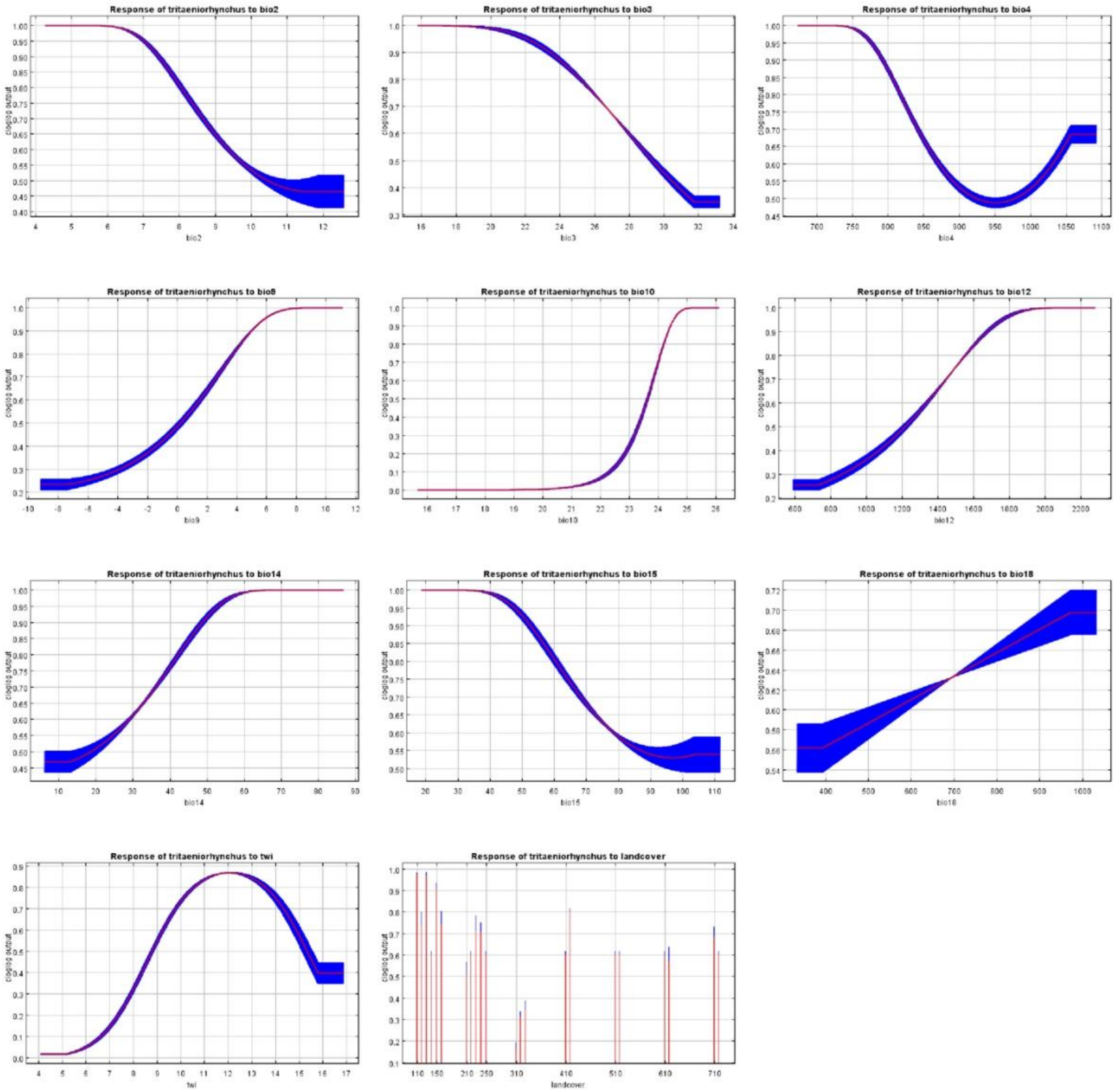

Figure S33. Response curves of Culex tritaeniorhynchus to environmental variables retained in the final MaxEnt model. Each panel shows predicted habitat suitability as a function of a single variable, with all other variables held at their mean values. See Table S1 for the variable set.

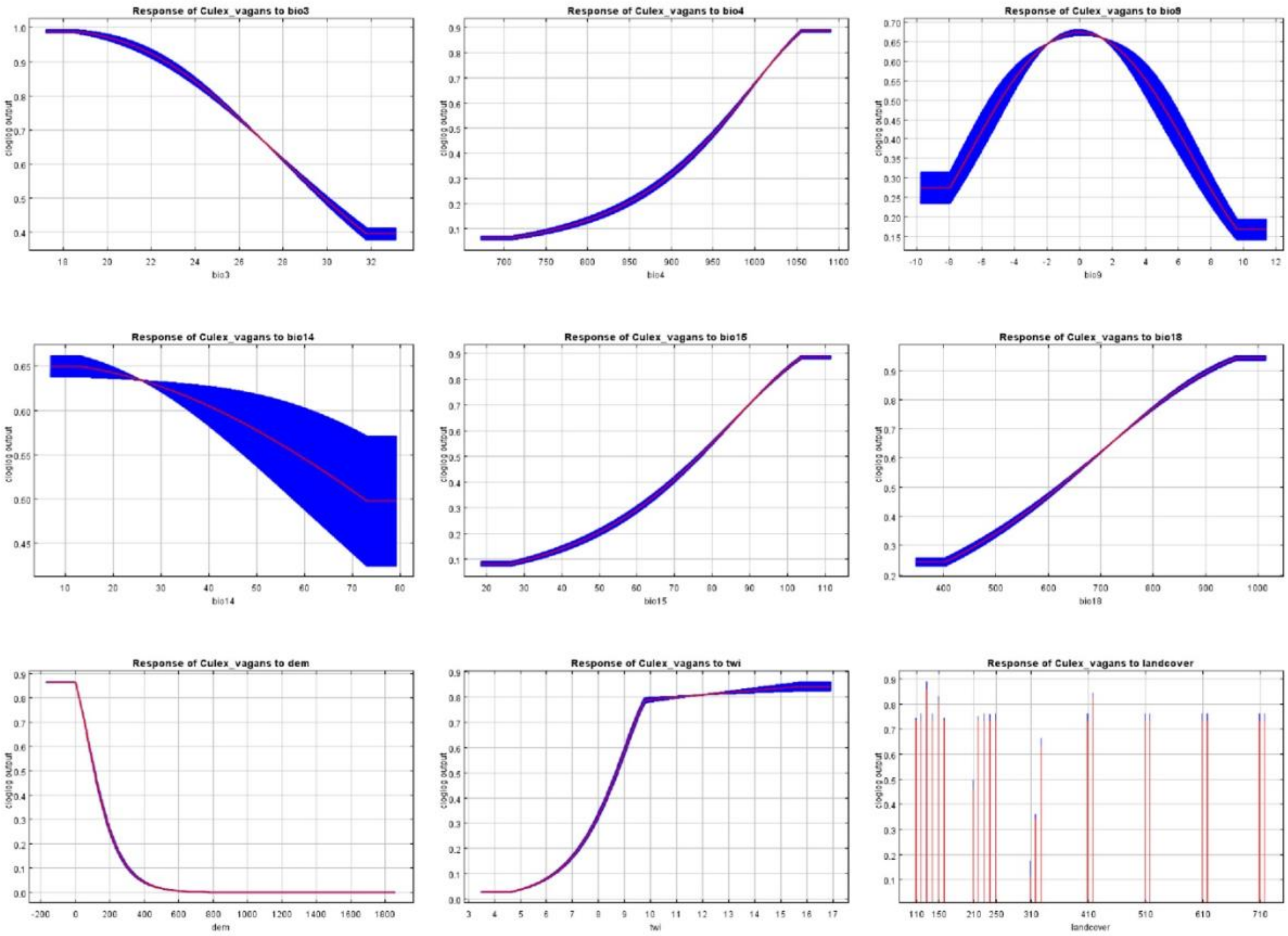

Figure S34. Response curves of Culex vagans to environmental variables retained in the final MaxEnt model. Each panel shows predicted habitat suitability as a function of a single variable, with all other variables held at their mean values. See Table S1 for the variable set.

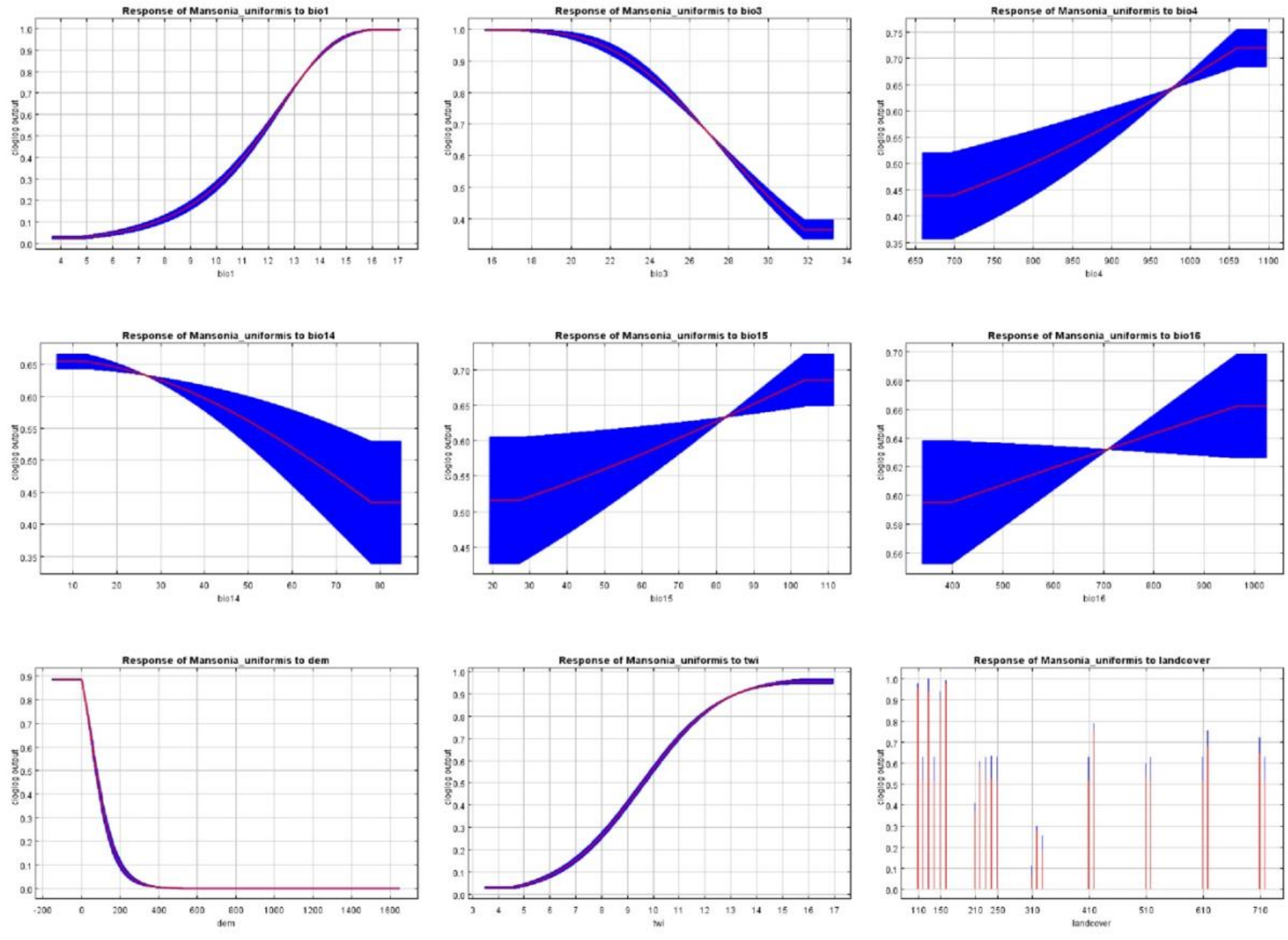

Figure S35. Response curves of Mansonia uniformis to environmental variables retained in the final MaxEnt model. Each panel shows predicted habitat suitability as a function of a single variable, with all other variables held at their mean values. See Table S1 for the variable set.

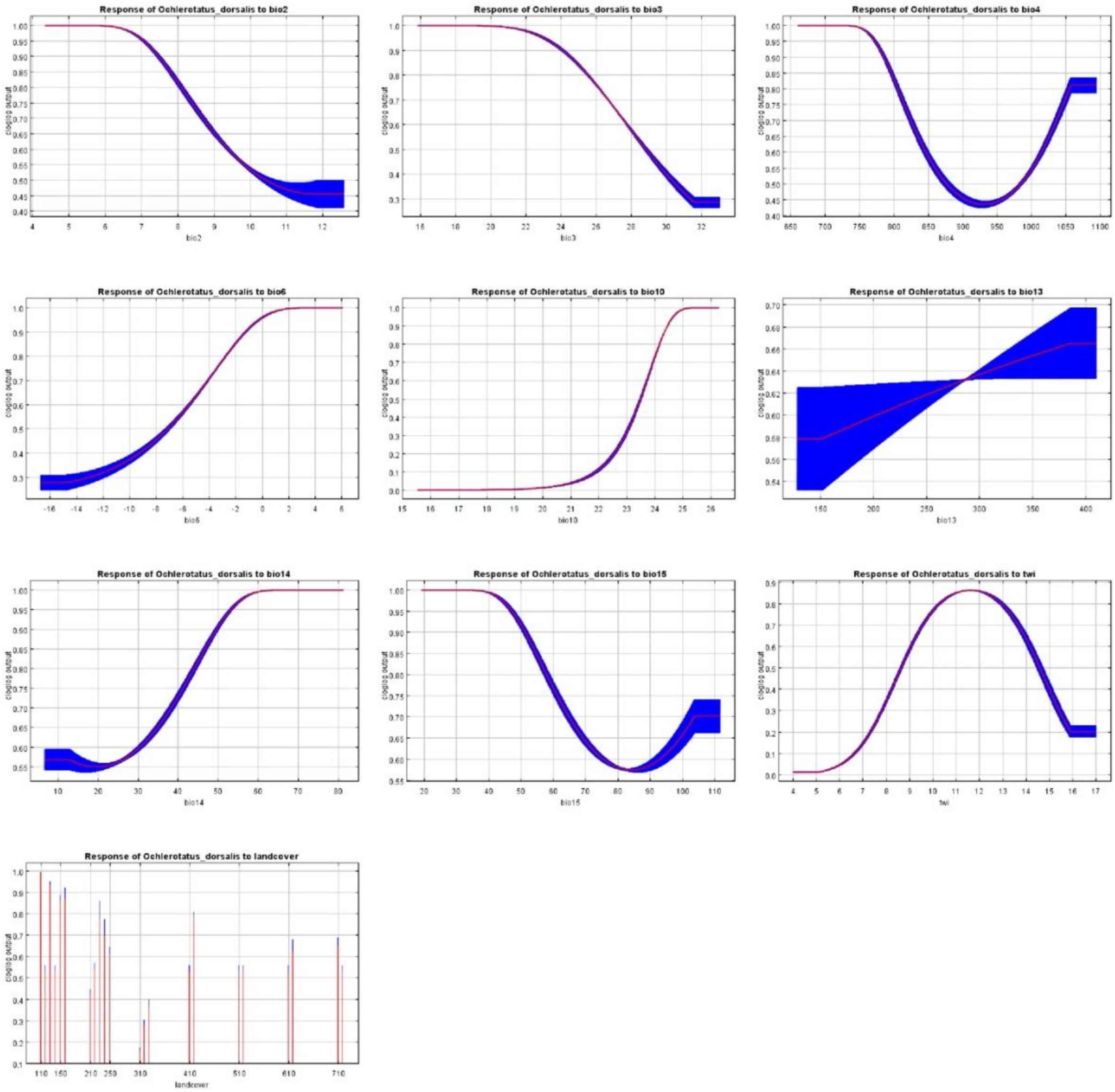

Figure S36. Response curves of Ochlerotatus dorsalis to environmental variables retained in the final MaxEnt model. Each panel shows predicted habitat suitability as a function of a single variable, with all other variables held at their mean values. See Table S1 for the variable set.

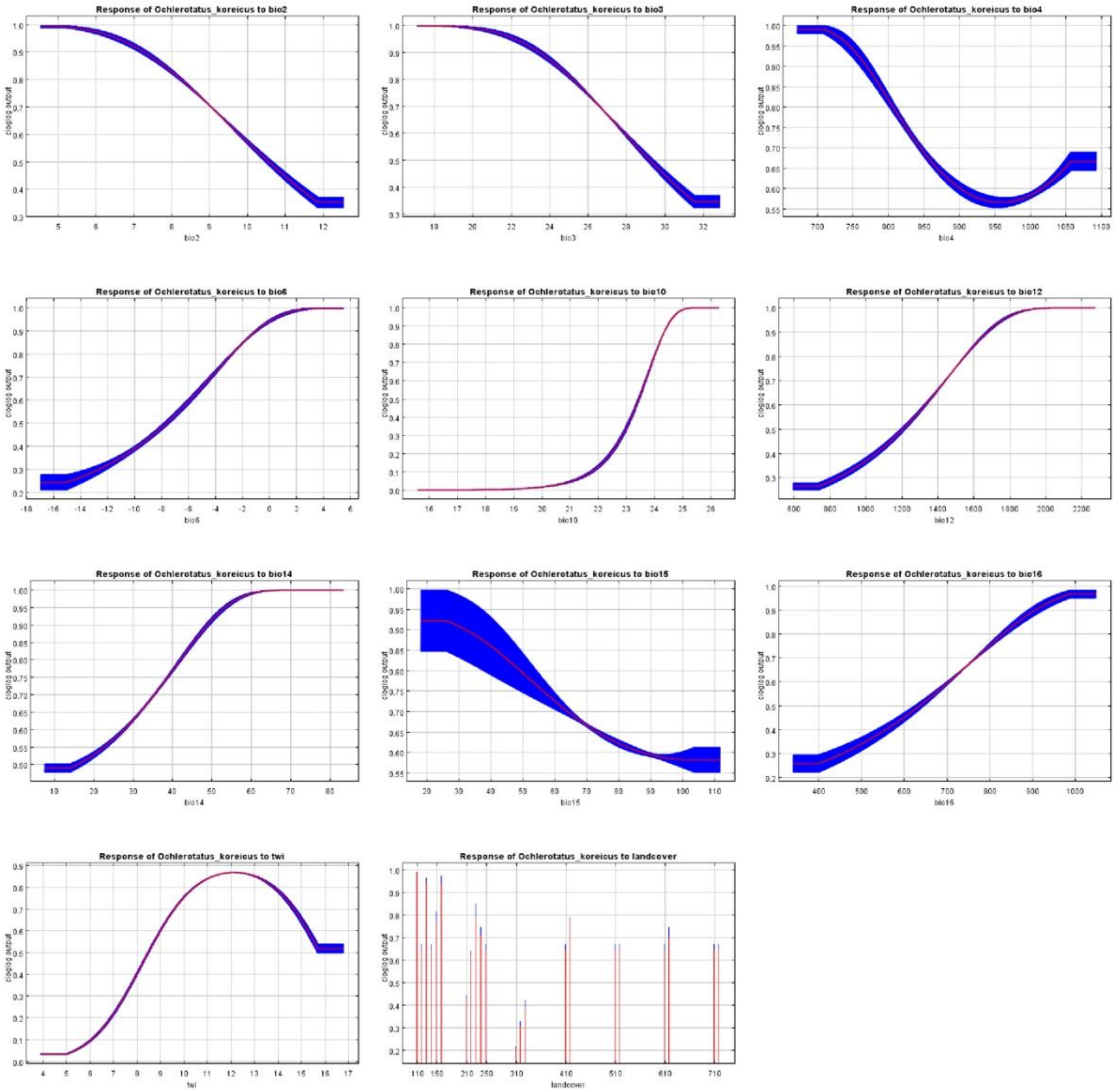

Figure S37. Response curves of Ochlerotatus koreicus to environmental variables retained in the final MaxEnt model. Each panel shows predicted habitat suitability as a function of a single variable, with all other variables held at their mean values. See Table S1 for the variable set.

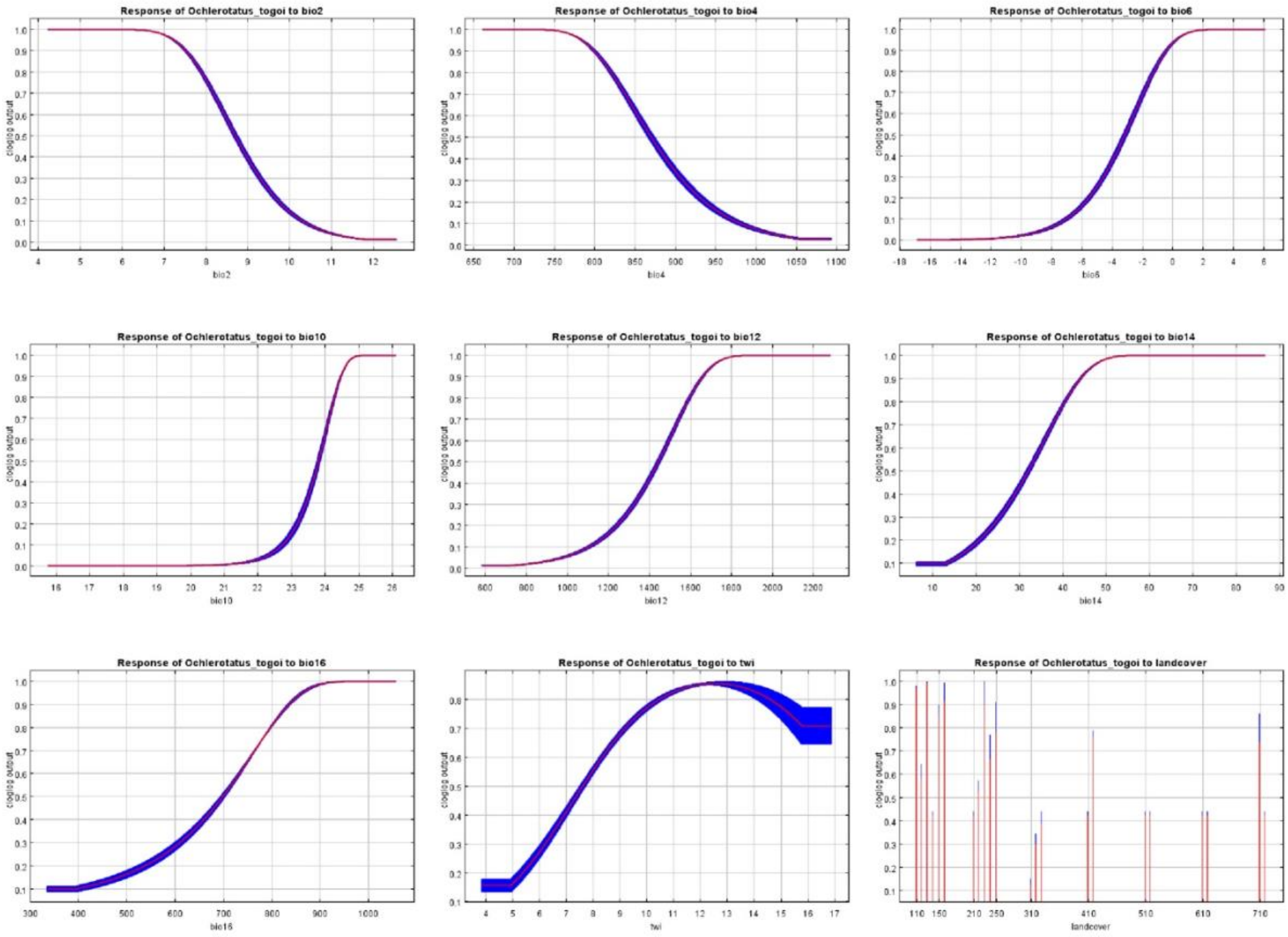

Figure S38. Response curves of Ochlerotatus togoi to environmental variables retained in the final MaxEnt model. Each panel shows predicted habitat suitability as a function of a single variable, with all other variables held at their mean values. See Table S1 for the variable set.
